# Decoding the regulatory landscape of lncRNAs as potential diagnostic and prognostic biomarkers for gastric and colorectal cancers

**DOI:** 10.1007/s10238-023-01260-5

**Published:** 2024-01-31

**Authors:** Arefeh Zabeti Touchaei, Sogand Vahidi, Ali Akbar Samadani

**Affiliations:** 1 Department of Chemistry, Lahijan Branch, Islamic Azad University, Lahijan, Iran; 2 Medical Biology Research Center, Kermanshah University of Medical Sciences, Kermanshah, Iran; 3 Guilan Road Trauma Research Center, Trauma Institute, Guilan University of Medical Sciences, Rasht, Iran

**Keywords:** Long noncoding RNA (lncRNA), Colorectal cancer, Gastric cancer, Drug resistance, Therapeutic targets

## Abstract

Colorectal cancer (CRC) and gastric cancer (GC) are major contributors to cancer-related mortality worldwide. Despite advancements in understanding molecular mechanisms and improved drug treatments, the overall survival rate for patients remains unsatisfactory. Metastasis and drug resistance are major challenges contributing to the high mortality rate in both CRC and GC. Recent research has shed light on the role of long noncoding RNAs (lncRNAs) in the development and progression of these cancers. LncRNAs regulate gene expression through various mechanisms, including epigenetic modifications and interactions with microRNAs (miRNAs) and proteins. They can serve as miRNA precursors or pseudogenes, modulating gene expression at transcriptional and post-transcriptional levels. Additionally, circulating lncRNAs have emerged as non-invasive biomarkers for the diagnosis, prognosis, and prediction of drug therapy response in CRC and GC. This review explores the intricate relationship between lncRNAs and CRC/GC, encompassing their roles in cancer development, progression, and chemoresistance. Furthermore, it discusses the potential of lncRNAs as therapeutic targets in these malignancies. The interplay between lncRNAs, miRNAs, and tumor microenvironment is also highlighted, emphasizing their impact on the complexity of cancer biology. Understanding the regulatory landscape and molecular mechanisms governed by lncRNAs in CRC and GC is crucial for the development of effective diagnostic and prognostic biomarkers, as well as novel therapeutic strategies. This review provides a comprehensive overview of the current knowledge and paves the way for further exploration of lncRNAs as key players in the management of CRC and GC.

**Graphical Abstract:**

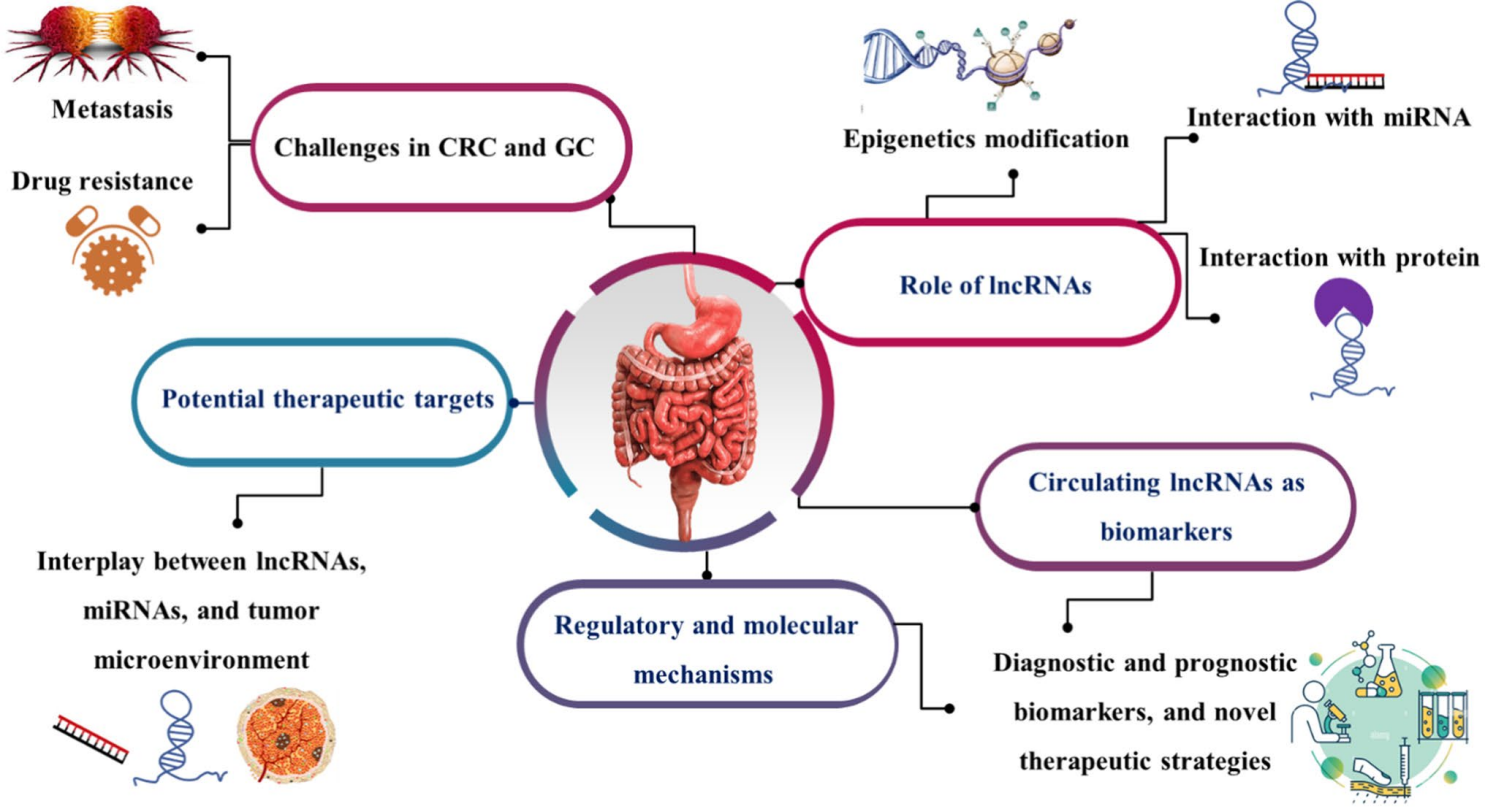

## Introduction

### Oncogenic and tumor suppressor lncRNAs

The well-researched oncogenic lncRNAs have a variety of targets and contribute in various ways to the development of tumors. Numerous studies have demonstrated that these lncRNAs are much more expressed in tumor tissues when compared to normal tissues, and individuals who express these lncRNAs more frequently have poor prognoses. It has been discovered that lncRNAs interact with numerous pathways to mediate oncogenic expression, which has a substantial impact on the appearance and development of various tumor cells [1]. Colorectal cancer-associated transcript (CCAT), H19, metastasis-associated lung adenocarcinoma transcript 1 (MALAT-1), and HOX transcript antisense intergenic RNA (HOTAIR) are among them; they are the most representative lncRNAs [2]. The earliest recognized long noncoding RNAs in CRC are called CCATs, and they are found at chromosome 8q24. The results of CCAT1 and CCAT2 showed how important lncRNAs are for CRC and stressed the demand for thorough lncRNA analysis in CRC [3]. The specific mechanism is that CCAL could cause multidrug resistance (MDR) by activating the Wnt/β-catenin signal pathway by inhibiting AP-2 and further enhancing MDR1/P expression (4). This suggests that CCAL could increase the development of CRC by targeting and activating AP-2, that CCAT1-L plays a part in the control of MYC transcription and encourages lengthy chromatin looping, and that CCAT1-L is site-specific transcribed upstream of MYC in human colorectal cancer. CCAT-1 may develop into a highly specific biomarker for CRC because it was elevated in both pre-malignant and malignant states in CRC. They demonstrated that CCAT2 could inhibit pre-miR-145 export to the cytoplasm and block the cleavage of premiR-145 by Dicer, demonstrating the new mechanism of lncRNA-miRNA in CRC. These findings suggest that CCATs may facilitate CRC advancement via a variety of pathways [5].

H19, which is expressed in opposition to IGF2 and is found on chromosome 11p15.5, IGF2 imprinting in the majority of human tissues is dependent on a differentially methylated region (DMR) upstream of H19. The expression of imprinted genes can be altered by abnormal DNA methylation, which may be one of the causes of CRC [6]. The mechanism by which CAFs could promote CRC stemness and chemoresistance by transferring H19 in exosome is that H19 could activate the β-catenin signaling by acting as a competing endogenous RNA sponge for miR-141, while miR-141 has the function of inhibiting CRC stemness. Studies have shown that H19 can promote the stemness of cancer stem cells (CSCs) and chemoresistance of CRC cells in CRC. That H19 may control the expression of CDK4 and CCND1, interact with the CDK8 gene transcription regulator macroH2A to control CDK8 expression, and influence β-catenin activity to advance CRC [7]. There was a strong correlation between the overexpression of the lncRNA H19 in CRC primary tumors and metastatic tissues with the disease's poor prognosis. By binding to nuclear hnRNPA2B1, which could increase RafERK signaling, ectopic H19 expression boosts CRC cells' metastasis and causes epithelial to mesenchymal transition (EMT) [8].

A lncRNA called MALAT-1 that is expressed from chromosome 11q13 was initially discovered to be overexpressed in non-small cell lung cancer. The significance of MALAT-1 in CRC metastasis; they examined the expression of MALAT-1 in five fragments and discovered that one fragment, which is positioned at the 3' end of MALAT-1 and affects CRC cell proliferation, migration, and invasion, is crucial. Furthermore, MALAT-1 was found to be substantially expressed in CRC tissues and may be a poor prognostic indicator in stage II and III CRC patients. Recent research has shown that MALAT-1 encouraged the growth, invasion, and metastasis of CRC [9]. MALAT-1 knockdown decreased the ability of CRC cells to transfer β-catenin between the cytoplasm and nucleus, which decreased the production of c-Myc and MMP-7 as well as their capacity for invasion and metastasis. These findings imply that metastasis-associated lung adenocarcinoma transcript 1 (MALAT1) controls the WNT β-catenin signaling pathway, which in turn controls CRC invasion and metastasis [10].

**Fig. 1:**
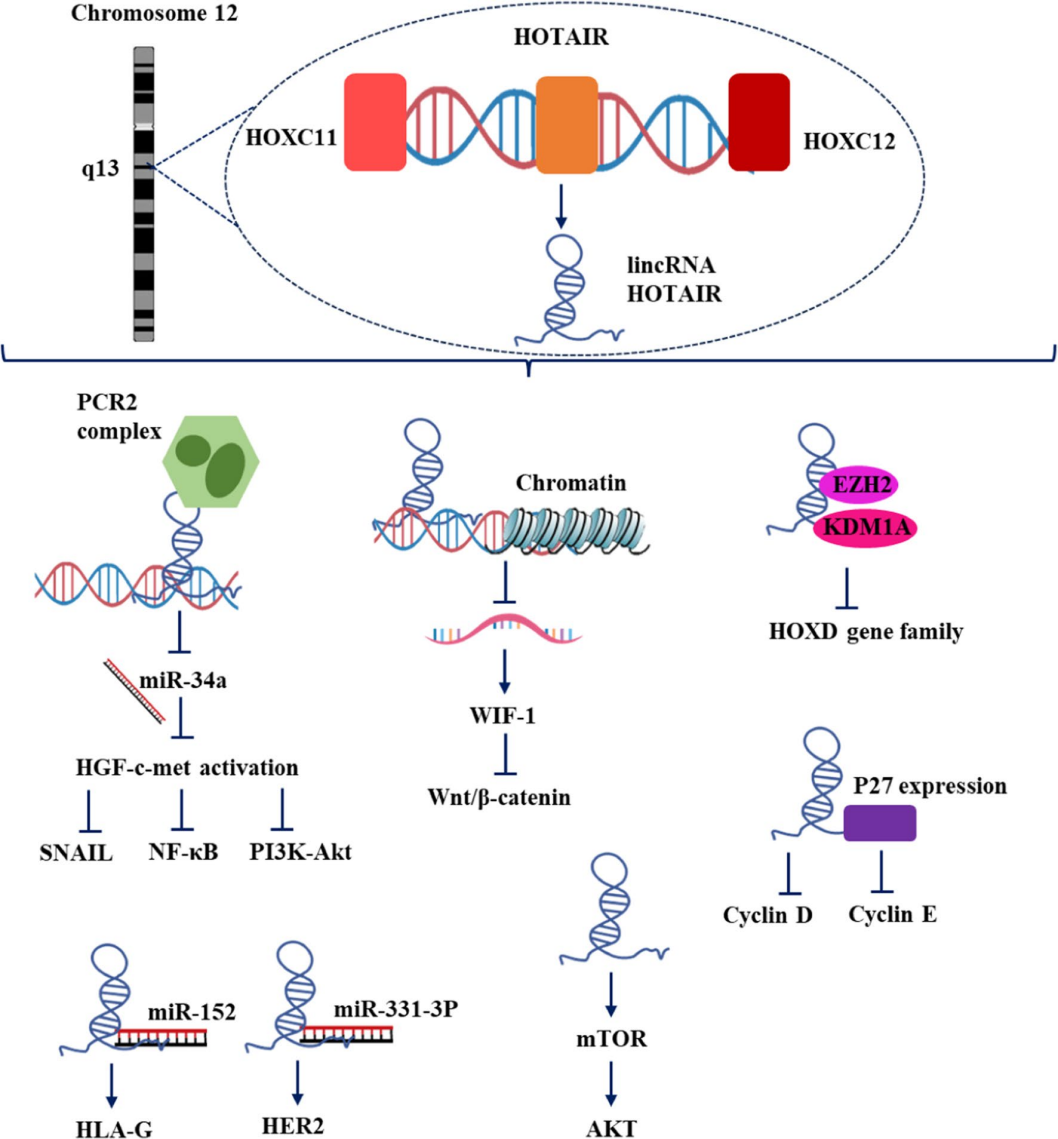
LincRNA HOTAIR regulation and function in GC and CRC

On chromosome 12q13.13, adjacent HOXC gene, HOTAIR is transcribed at the HOXC locus. The level of HOTAIR expression was higher in CRC tissues than in healthy tissues, and cDNA array analysis revealed a strong correlation between HOTAIR expression and PRC2 complex (SUZ12, EZH2, and H3K27me3). HOTAIR increased CRC progression by promoting lung and liver metastases and treatment resistance (Fig. 1) [11].

The tumor suppressors RB and p53, as well as the antigen peptide-loading complex, are degraded by the oncogene lncRNA LINC01139, one of the abnormally expressed proteins in the antigen presentation system that contributes to immune escape. Another well-known example is the oncogenic lncRNA PCAT19, which aids in the growth of tumor cells in prostate cancer, induces the expression of MYC, a transcription factor, and lessens the likelihood that damaged DNA strands will be repaired by suppressing the expression of the breast cancer 2 genes. It is interesting to note that MYC shares similarities with the lncRNA gene Plasmacytoma Variant Translocation 1 (PVT1), an oncogene that is overexpressed in malignancies. It has been demonstrated that p53 induces the alternative transcript PVT1B during genotoxic stress, which helps p53-mediated tumor growth restriction in vivo by regulating the transcription of the nearby MYC genes. Alternatively, it has also been shown that the lncRNA LINC02525, which is upregulated in neuroblastomas and interacts with the ribosomal protein RPL35 to activate the translation of E2F1, maintains the stability of the NMYC protein, and causes ERK protein phosphorylation, plays an obvious oncogenic role [12, 13].

PTEN is a well-known tumor suppressor that plays a critical role in the development and spread of numerous malignancies. PTEN-dependent signal pathway demonstrated that Linc02023 reduced CRC carcinogenesis via controlling PTEN stability. One study discovered that LINC01559 was downregulated in CRC tissues compared to normal tissues, and lower expression of LINC01559 in CRC patients indicated a poor prognosis; this study also revealed a novel mechanism of METTL3-mediated m6A modification on LINC01559 as the mechanism of CRC progression [14].

One of the key mechanisms of CRC metastasis is EMT. Tumor suppressor lncRNAs can prevent the EMT process in CRCs by acting on a variety of targets. As a result, lncRNAs linked to EMT are receiving more attention. SRSF6 has been shown to promote EMT and metastasis in CRC cells, while LINC01133 inhibits these processes by acting on SRSF6. Additionally, lncRNA SATB2-AS1 expression was correlated with tumor staging and prognosis in normal tissues and drastically decreased in CRC tissues. Additional research revealed that SATB2-AS1 overexpression prevented CRC cells from proliferating, migrating, or invading in both vivo and in vitro. Another study found that SATB2-AS1 could directly bind to WDR5 and GADD45A, mediate the deposition of H3K4me3 in the SATB2 promoter region and DNA demethylation, and regulate the expression of Th1-type chemokines and immune cell density in CRC tissues. These actions activated SATB2 transcription [15].

The expression of the lncRNA MIR4435-2HG in CRC tumor tissues was shown to be considerably higher than that in normal tissues in 2022, according to data from the GEO and TCGA databases. Additional research revealed MIR4435-2HG to be a tumor suppressor gene that controls the immune microenvironment. Reduced neutrophils and increased polymorphonuclear myeloid-derived suppressor cells (PMN-MDSCs) were the results of MIR4435-2HG deletion. MIR4435-2HG deletion in neutrophils has been shown to increase the immunosuppressive potential of PMN-MDSCs by interfering with their fatty acid metabolism and accelerating the development of colorectal cancer in tissue-specific MIR4435-2HG knockout mice [16].

lncRNA DIRC3's expression level is inversely connected with patients' shorter survival rates in melanoma, and subsequent research has revealed that it can trigger the transcription of the tumor suppressor IGFBP5 by altering the local chromatin structure. A large number of lncRNAs are also part of the tumor suppressor system that is initiated by p53, a transcription factor well known for maintaining the homeostasis of the cell. Some of these p53-regulated lncRNAs are downregulated in CRC, indicating their tumor-suppressive functions [17].

Maternally expressed gene 3 (lncRNA MEG3) is one of these tumor suppressor lncRNAs that have received a lot of attention [13]. Researchers have demonstrated that the lncRNA MEG3 could control p53 expression to act as a tumor suppressor gene. In meningioma and NSCLC, MEG3 may control cell growth and death by activating p53. Additionally, through controlling the expression of p53, the lncRNA MEG3 may prevent the spread of liver cancer [18]. The relationship between lncRNA MEG3 and p53 in gastric cancer is still mostly unknown; however, MEG3 may function as a tumor suppressor gene in this disease. In gastric cancer tissue, lncRNA MEG3 expression was decreased. Significantly, MEG3's expression in gastric cancer tissue was correlated with the size of the tumor. The ability of gastric cancer cells to proliferate and metastasize may be decreased by the suppression of the lncRNA MEG3. Finally, by controlling the expression of p53, the lncRNA MEG3 could cause suppression in gastric cancer [19].

The lncRNA LINC00261 participates in the DNA damage response in lung cancer and can inhibit proliferation by inducing G2-M cell cycle arrest. It has been shown that its expression is significantly lower than normal tissue samples in various cancers, including liver, breast, and gastric cancer. Additionally, lncRNA's inhibitory influence can be seen in the regulation of signaling [20]. For instance, the decreased expression of DRAIC in castration-resistant advanced prostate cancer can stop the disease's growth by preventing nuclear factor-B (NF-B) from being activated by interfering with the activity of its kinase inhibitor, IKK. It limits the interaction between IKK and the IKK complex subunits, which prevents NF-B from being activated [21].

**Fig. 2:**
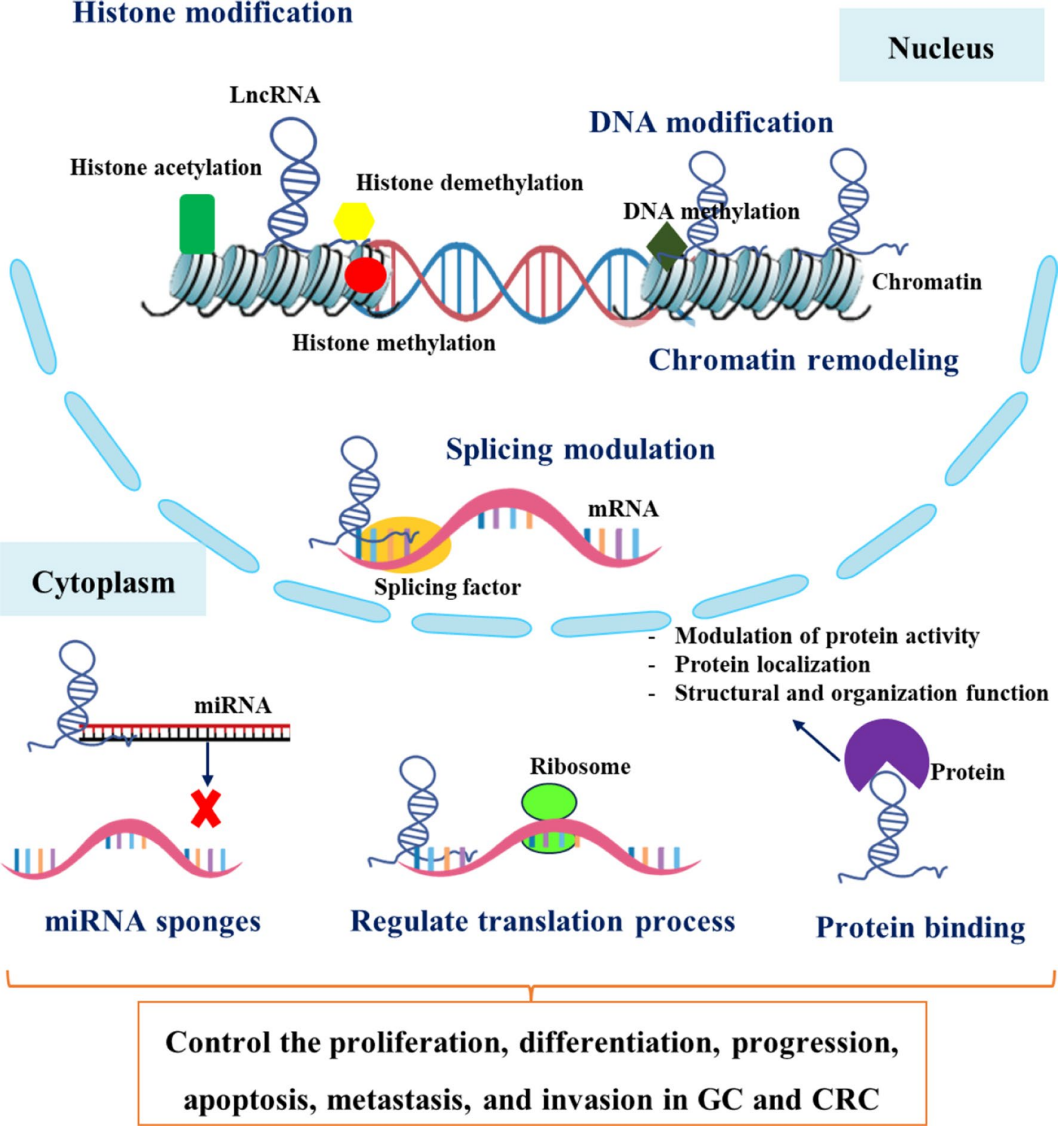
LncRNAs function in GC and CRC. In the nucleus, lncRNAs participate in epigenetic modifications including histone modifications (histone acetylation, histone methylation, histone demethylation), chromatin remodeling, DNA methylations, and splicing modulations of mRNAs. In the cytoplasm, lncRNAs can interact with RNAs including miRNA sponges and regulate translation. Moreover, lncRNAs bind to proteins consisting of modulation of protein activity, protein localization, and structural and organization function

LncRNAs are essential in the emergence of cancer. LncRNAs regulate gene expression through various mechanisms, including epigenetic modifications and interactions with microRNAs (miRNAs) and proteins (Fig. 2). Since epigenetic and genomic alterations play a crucial role in controlling lncRNA expression in tumors, these lncRNAs have the ability to influence both protein-coding and noncoding genes as well as interact with other cancer-related genes. Different characteristics of lncRNAs can operate as tumor suppressors, oncogenes, or possible therapeutic agents [22, 23]. It is believed that the fundamental reason for these variances is due to their complex structures and capacity to participate in multicomponent complexes. LncRNAs play both tumor suppressor and oncogenic roles in different forms of cancer. They differ in gene expression unique to a tissue or cell type. Colorectal tumors have been found to have high levels of KCNQTOT1 lncRNA transcription; however, its exact function is yet understood. It has been demonstrated that lncRNA ANRIL may influence carcinogenesis in a number of cancer types [24]. Also, several studies have suggested that an increase in ANRIL promotes the progression of nasopharyngeal carcinoma and, consequently, tumor development. According to other studies, HOTAIR functions as a trans-acting lncRNA and is linked to stomach cancer. According to a publication, overexpression of HOTAIR may encourage the growth of cancer cells in tissues by causing metastasis to spread to additional sites and organs [25].

It was revealed that CRC HT-29 cell invasion, migration, and proliferation may be inhibited by the overexpression of GAS5. Also, overexpression of GAS5 during apoptosis caused colorectal cancer cells to proliferate [26].

A potential new biomarker and therapeutic target for GC is the oncogenic lncRNA WFDC21P, which accelerates tumor growth through the WFDC21P/Ran/Akt/GSK3/ β-catenin axis and is favorably controlled by transcription factor FOXP3 [27].

Numerous lncRNAs play a crucial role in numerous biological processes in GC. Abnormal DNA methylation in gastric cancer affected a number of lncRNAs that served as tumor suppressor genes [28]. For instance, the transcribed ultraconserved regions (T-UCRs) produced by Uc.160, one of the ultraconserved regions (UCRs), have CpG islands on their upstream encoding region, where the hypermethylation is cancer specific. According to a study, Uc160 + was suppressed in GC samples compared to normal stomach samples due to hypermethylation of CpG islands upstream of Uc.160 + , which is linked to the growth of tumor cells and ultimately results in tumor proliferation [29]. Further research was carried out to determine the regulatory mechanisms, and it was hypothesized that Uc.160 + might plausibly be involved in the activation of the MAPK signaling pathway, which results in the inactivation of runt-related transcription factor 3 (RUNX3) by invoking the Src/MEK/ERK and p38 pathway, and contribute to the repression of PTEN expression (30). Also, the expression of small integral membrane protein 10 like 2A (linc00086) was lower in GC tissues compared to normal tissues because DNA methylation was controlling MeCP2 (Methyl-CpG-binding protein 2) via ERK1/2 signaling pathways. MeCP2 is similarly involved in the progression of cancer by binding methylated CpG islands. Linc00086 may be expressed when MeCP2 is silenced, which may indicate that DNA methylation is related to the downregulated expression of Linc00086 in GC [31].

Another lncRNA, known as AFDN-DT (AFDN divergent transcript), similarly functions as a tumor suppressor in GC and was found to be present at low levels in GC patients. Additionally, one of the primary mechanisms for the downregulation of AFDN-DT was thought to be hypermethylation of the AFDN-DT promoter, which allowed GC to proliferate and metastasize in xenografts in vivo [32]. The existence of SPRY4-IT1, a long-coding RNA derived from an intron inside the SPRY4 gene, which was first identified in melanoma cells as a tumor suppressor, has been demonstrated in numerous other cancer cells, including kidney cancer and esophageal cancer. It was intriguingly suggested that SPRY4-IT1 was severely depleted in GC cells, causing proliferation, invasion, and metastasis in vitro and in xenograft vivo as a result of the epithelial-mesenchymal transition, which was attributed to a decrease in E-cadherin and an increase in vimentin primarily. Additionally, SPRY4-IT1 promoter region was highly hypermethylated, which limits its expression (33).

### lncRNA controls the expression of genes at transcriptional and post-transcriptional levels

According to previous research, it has been found several lncRNA works together with multiple histone-modifying complexes. This results in DNA methylation and chromatin modification, which ultimately leads to the silencing of the target mRNA expression. For instance, the sequencing found that only HOXA11-AS was specifically over-expressed in GC and not in other malignancies. Patients with GC with high levels of HOXA11-AS had a bad prognosis. The reduction in HOXA11-AS expression greatly decreases the proliferation of gastric cancer cells and enhances the occurrence of apoptosis. It appears that HOXA11-AS is more commonly found in the nucleus [34]. Given that HOXA11-AS can directly connect to the RNA-binding proteins LSD1, PRC2, and DNMT1, also it functions as a scaffold to decrease the transcriptional expression of KLF2 and PRSS8. Finally, PRC2 and LSD1 are targeted for linked H3K27 methylation and H3K4 demethylation by the lncRNA-mediated assembly of PRC2 and LSD1 [35]. Additionally discovered that GC had much higher levels of the GClnc1 (gastric cancer-associated lncRNA1). In a manner that changes GC proliferation, migration, and invasion, GClnc1 binds to WDR5 and KAT2A histone acetyltransferase, functions as a modular scaffold of WDR5 and KAT2A complexes, and controls their distribution. On the other hand, it was discovered that individuals with GC who had low expression of FENDRR (fetal-lethal noncoding developmental regulatory RNA) had a poor prognosis. FENDRR overexpression can drastically lower the metastasis of GC [36]. FENDRR and PRC2 interact to generate the FENDRR/PRC complex, which prevents the expression of MMP2/9. In order to accelerate the development of GC, it has been discovered that lncRNA epigenetically silences miRNA expression at the transcript level. HOTAIR is located at the HOXC locus. It modifies cell epigenetics and gene expression through a trans interaction with polycomb repressive complex 2 (PRC2) [36]. The overexpression of HOTAIR in cancer epithelial cells was discovered to cause histone methylation and cancer invasion. Additionally, it is expressed in a manner that is strongly related to a number of proteins, including vimentin, matrix metalloproteinase, and E-cadherin, all of which are implicated in the invasion, metastasis, tumor stage, and angiogenesis of CRCs. Not only in CRC but also in other malignancies such as gastric, breast, pancreatic, and human epithelial ovarian cancers, as well as in hepatocellular carcinoma, HOTAIR expression has been associated with metastasis and a poor overall prognosis [37]. According to CRC blood and tissue samples, overexpression of HOTAIR is associated with a high patient mortality rate and can be used as a prognostic marker for sporadic CRC. Moreover, the overexpression of HOTAIR was linked to the development of GC and was associated with a poor prognosis, according to earlier research. HOTAIR joined with EZH2 and SUZ12 to create a complex that binds to the miR-34a promoter directly and modifies H3K27me3 to inhibit the expression of the miRNA [38]. The decreased miR-34a expression greatly silences HGF/c-met activation, which triggers PI3K/Akt, SNAIL, and NF-kB signaling to promote cancer growth. lncRNA overexpression ANRIL has a strong correlation with the advancement of GC and can be used as a standalone predictor of patient survival. E2F1 directly attaches to the ANRIL promoter to cause its expression. Then, high levels of ANRIL in combination with PRC2 dramatically suppress miR-99a/miR-449a expression at the transcriptional level, leading to an increase in the expression of mTOR, CDK6, and E2F1. To prevent mTOR, E2F1, and CDK6 from being expressed, miR-99a and miR-449a bind to their 3′UTRs. ANRIL expression was generally induced by increased E2F1 expression (39). The ANRIL/PRC2 complex induced mTOR, CDK6, and E2F1 expression while suppressing miR-99a/miR-449a expression. Furthermore, E2F1 increased the expression of ANRIL, creating a positive feedback loop that aided in the growth of GC cells. By directly interacting with the target gene's mRNA, lncRNAs also control the expression of the gene [40]. The promoter of the lncRNA TINCR is bound by the high expression of nuclear factor SP1, which is considerably elevated in gastric cancer, and thus promotes the expression of the lncRNA. The proliferation, tumorigenicity, and apoptosis of cancer cells are all considerably reduced when TINCR expression is silenced. Mechanistic investigations revealed that the majority of TINCR was found in the cytoplasm [41]. The combination of TINCR and STAU1 serves as a staufen-mediated mRNA decay (SMD) factor, as demonstrated by RNA IP and pull-down test. Additionally, it was demonstrated by RNA immunoprecipitation (RIP) and RNA pull-down assay that TINCR/STAU1 complexes directly bind with KLF2 mRNA, reduce KLF2 mRNA stability, and impede the expression of KLF2 protein. Finally, increased KLF2 expression boosts cancer cell proliferation, migration, invasion, and tumorigenicity by decreasing CDKN2B/P15 and CDKN1A/P21 transcripts [42]. As opposed to healthy controls, GC patients had significantly higher levels of GHET1 than normal individuals. In vitro, the overexpression of GHET1 greatly enhances cell proliferation in MKN45 and AGS cell lines. The formation of xenograft tumors in naked mice was dramatically accelerated by the over-expression of GHET1 in MKN45 cells, according to in vivo tests. The lower RIP and RNA pulldown assay originally demonstrated mechanistically that GHET1 may directly connect with insulin growth factor 2 binding protein 1 [43]. RIP assay and qRT-PCR demonstrated that IGF2BP1 interacted with the c-myc mRNA. These findings have collectively shown that the lncRNA GHET1/IGF2BP1 complex interacts with c-myc mRNA to improve its stability and encourage protein expression. Moreover, increased c-myc expression aided GC cell proliferation [44].

MiRNA controls target gene expression at the post-transcriptional level, as was previously mentioned. As a result, lncRNAs can be categorized as competing endogenous RNAs (ceRNAs), which function as miRNA sponges to control the migration, invasion, and proliferation of GC and CRC cells by inhibiting the targets of miRNAs. lncRNAs work with miRNA like a sponge and trigger a "ceRNA" to control gene expression. The expression of hTERT is inhibited by the combination of miR1207-5p and the 3′UTR, which dramatically reduces the proliferation and metastasis of cancer cells [45]. In GC, BC032469 significantly promotes cell proliferation and metastasis and its expression was positively correlated with hTERT expression. BC032469 directly binds to miR-1207-5p, as proven by RIP and Northern blot. So, BC032469 may be a poor prognostic marker for GC because it acts as a ceRNA to inhibit miR-12075p-dependent hTERT downregulation. In addition to the transcriptional; lncRNA controls protein stability at the post-transcriptional level to advance GC [46]. The transcriptional factor FOXM1 has binding sites in the promoter of PVT1 which is dramatically increased in GC with high FOXM1 expression. Furthermore, whereas FOXM1 protein can reversely bind to PVT1 to boost stability and prevent 26s proteasome-mediated degradation, high expression of PVT1 did not affect FOXM1 mRNA expression. As a result, high levels of FOXM1 and PVT1 expression create a positive feedback loop that encourages the growth and metastasis of GC [47].

Some lncRNAs have a tendency to be overexpressed in CRC cells and A lncRNA called MALAT1, or metastatic-associated lung adenocarcinoma transcript 1, interacts with pre-mRNA to locate transcriptionally active genes in chromatin and with serine/arginine splicing factor to control alternative splicing. MALAT1 activates AKAP-9, a gene associated with the growth and metastasis of various malignancies, including oral cancer, melanoma, breast, thyroid, and lung cancer. This activation of AKAP-9 occurs in CRC tissues and cells and increases cancer cell proliferation, invasion, migration, and lymph node metastasis. By encouraging the expression of SRPK1 and phosphorylating SRSF1 in CRC cells, MALAT1 increases the expression of AKAP-9 [48].

In H19 on chromosome 11p15.5, there is the H19 lncRNA. Only during the early stages of embryogenesis H19 is elevated and then downregulated. The transcription of the H19/insulin-like factor 2 gene cluster led to the discovery of H19, which is expressed by the maternal allele rather than the paternal allele. H19 controls the expression of several cancer-related proteins, including the retinoblastoma tumor suppressor, ubiquitin ligase E3 family, and calneuron 1. Studies show that the dysregulation of H19 has a variety of impacts on many malignancies, including CRC. Differentially methylation region of H19 and its upstream of exon 3 of IGF2 were hypomethylated in CRC tissues and normal mucosa. Poor differentiation and a high TNM stage are linked to the overexpression of H19 in CRC tissues [49].

The oncofetal gene UCA1 (human urothelial carcinoma-associated 1), which controls embryonic development, was initially expressed in bladder cancer and CRC tissues, which contributes to cell proliferation by enhancing CRC carcinogenesis and blocking apoptosis, according to recent studies. UCA1 serves a variety of biological roles in cancer cells, including promoting cell proliferation and transformation, increasing invasion and mortality, and causing treatment resistance in cancer cells. Since UCA1 is overexpressed in CRC tissues, silencing it could aid in reducing the progress of the disease. Furthermore, increased UCA1 expression may lead to larger tumors and deeper tumors [50].

On chromosome 8q24, there is a gene known as CASC11 whose expression in CRC patients was discovered to be linked with tumor growth, and it is elevated in CRC cells and tissues. Additionally, the suppression of CASC11 in CRC inhibits the spread and proliferation of tumor cells [51].

In CRC tissues, it was discovered that 90% of colorectal neoplasia differentially expressed (CRNDE) was overexpressed. It has been demonstrated that insulin and IGFs control CRNDE through metabolic adjustments to cause cancer cells to exhibit the Warburg effect. A recent study found that CRNDE-h was substantially expressed in CRC tissues and that its expression was associated with tumor size, distant metastasis, a low overall survival rate, and lymph node metastasis [52]. Additionally, CRNDE levels in CRC tissues were increased, and this overexpression was directly linked with tumor size and stage. Additionally, CRC cells may have an increase in in-vitro and in-vivo apoptosis if CRNDE is knocked down. By blocking the Wnt/ β-catenin signaling pathway, CRNDE and miR-181a-5p microRNA knockdown lowered chemoresistance and suppressed cell proliferation [53].

PCAT-1 (prostate cancer-associated ncRNA transcript 1) was initially discovered in prostate cancer and may also be related to CRC metastasis. By encouraging PRC2 expression, PCAT-1 overexpression in cancer cells was linked to an increase in in vitro cell proliferation. PCAT-1 was discovered to be overexpressed in non-small-cell lung cancer tissues and cells, where it increased cell proliferation, migration, and invasion. Additionally, a study found that PCAT-1 was overexpressed in CRC tissues and that this overexpression was associated with distant metastases. Intriguingly, the expression of PCAT-1 in colon tissues was substantially linked with CRC patient survival, and patients with high PCAT-1 expression had a shorter survival rate than those with lower PCAT-1 expression. A recent study found that downregulating PCAT-1 in CRC cells decreased cell proliferation and stopped cell cycle transition by decreasing the expression of cyclins and c-myc and that PCAT-1 expression in CRC cells is closely linked with c-myc [54].

lncRNA strongly interacts directly with the promoter and controls gene expression in the cytoplasm and nucleus. The polycomb group protein (PcG) complex in the nucleus is bound by lncRNAs, which also cause histone trimethylation and control transcriptional regulation of relative gene mRNA expression (55).

### lncRNAs in GC and CRC development and progression

The prolonged growth and resistance to apoptosis of cancer cells are their most defining characteristics. These biological functions of GC cells entail intricate signaling pathways as well as several other elements. The intricate mechanisms, however, remain unresolved. Numerous oncogenic lncRNAs, including SNHG7, CCHE1, and lncMIF-AS1, have been found to encourage GC cell growth and prevent apoptosis [56]. In contrast, MT1JP and BG981369 are downregulated as the GC progresses which have anti-tumor properties, which are demonstrated by the fact that they increase apoptosis and reduce proliferation in GC cells when overexpressed. The GC cell line SGC7901's viability is decreased and its proportion of apoptotic cells is increased when the lncRNA AFAP1-AS1 is knocked down. Further research showed that AFAP1-AS1 regulates the proliferation and apoptosis of GC cells by focusing on the PTEN/p-AKT signaling pathway, as evidenced by the finding that AFAP1-AS1 knockdown decreases the expression of p-AKT and increases the expression of PTEN [57]. By controlling the MAPK signaling pathway, Linc00483 and AOC4P have been demonstrated to have an impact on the proliferation and death of GC cells. Additionally, it has been demonstrated that several lncRNAs, including ZEB2-AS1 and TOB1-AS1, influence proliferation and apoptosis in GC development in a fashion that is dependent on the Wnt/ β-catenin signaling system [30]. The pathogenic processes that are most directly linked to GC patients' mortality are invasion and metastasis. It is understood that invasion is the first stage of metastasis. According to research, lncRNAs have a key role in controlling invasion and metastasis in GC. As an example, LINC00163 targets the miR-183/AKAP12 axis and functions as a ceRNA to prevent the invasion and metastasis of GC cells [58]. LncRNA PCGEM1 stimulates miR-129-5p to boost the production of P4HA2, which aids in the invasion and metastasis of GC cells. We showed in our earlier research that the lncRNA GCRL1 is increased in GC tissues and cell lines. While GCRL1 knockdown greatly reduces the number of migratory and invasive cells, GCRL1 overexpression significantly increases the number of invasive GC cells [59]. The study also demonstrated that, in a mouse lung metastasis model, the knockdown of GCRL1 reduces the metastasis of GC cells. Additionally, it was discovered that miR-885-3p sponging by GCRL1 facilitates invasion and metastasis in GC cells. It has been demonstrated that the abnormal activation of EMT gives cancer cells the ability to move and invade. lncRNAs like PCGEM1 control invasion and metastasis in GC cells via promoting EMT, according to mounting data. The MALAT-1 is knocked down, which prevents GC cell invasion and migration [60]. Other research has demonstrated that MALAT-1 knockdown increases the expression of E-cadherin while decreasing the expression of the vimentin-associated EMT marker. Additionally, the features of lncRNAs in GC progression may be partially explained by their role in cell cycle process regulation. An essential intracellular modulator involved in the control of the cell cycle is cyclin-dependent kinase 4 (CDK4). Furthermore, it has been noted that the suppression of the lncRNA HOXA11-AS causes GC cells to enter a G0/G1 phase arrest and prevents GC cells from migrating, invading, and metastasizing [9]. HOXA11-AS controls GC development by influencing the expression of P21, KLF2, cyclin D1, and CDK2 as well as the transcription of β-catenin. It has been demonstrated that lncRNAs regulate the expression of numerous essential genes involved in the advancement of the cell cycle, including CDK1, CDK6, and cyclin E1. These results suggest that lncRNAs regulate the expression of unique genes implicated in several signaling pathways to exercise their oncogenic or anti-tumor effects in GC [61].

The multi-step etiology of CRC is fueled by genetic and epigenetic changes that disturb cellular function. These changes set in motion an evolutionary process that is primarily characterized by the development of hallmark abilities for transforming CRC cells, such as maintaining proliferative signaling, resisting apoptosis, enabling replicative immortality, and activating EMT program, angiogenesis, invasion, and metastasis [62]. The deregulation of a lncRNA can affect the phenotypic alteration of multiple tumor processes at the same time, including the induction of proliferation, invasiveness, and metastasis of tumor cells, which is relevant given the role of some lncRNAs in the initiation and progression of CRC [63].

Further insights into the pathogenesis of CRC can be gained by understanding the role of lncRNAs in tumorigenic signaling pathways like Wnt/ β-catenin, EGFR/IGF-1R, KRAS, phosphatidylinositol-3-kinase (PI3K), transforming growth factor-beta (TGF-), p53, and EMT signaling pathways [64].

The lncRNA regulator of reprogramming (lnc-ROR), which promotes the growth, invasion, migration, and drug resistance of numerous cancer cells such as lung cancer, hepatocellular carcinoma, breast cancer, and CRC cells, is known to control the development of different cancers. Lnc-ROR sponges miRNAs in CRC cell lines that control stem cell factors such POU class 5 homeobox 1, Nanog, and SRY-box 2, and it also reduces radiation sensitivity by downregulating the p53/miR-145 pathway. Apoptosis-related protein is inhibited by miR-6833-3p, which is downregulated by overexpressed lnc-ROR. This is related to the activation of the EMT pathway and metastasis in CRC [65].

Deregulation of lncRNA expression can place in a tissue- or organ-specific manner and seems to be closely linked to the development and onset of CRC [66]. Long QT intronic transcript 1 (LIT1/KCNQ1OT1) loss of imprinting has been reported to be correlated with deregulation of a lncRNA in CRC, suggesting its potential as a helpful diagnostic for CRC diagnosis [67]. HOTAIR binds to lysine-specific histone demethylase 1A (KDM1A) and polycomb repressive complex 2 (EZH2) in the 5′ and 3′ regions, repressing the transcription of the homeobox D cluster (HOXD) family genes in the progression of breast cancer and CRC. In CRC patients, distant metastases and a poor prognosis are highly linked with high HOTAIR expression, and it has been identified as a circulating biomarker in the blood of CRC patients as well as being validated as a poor prognostic factor in original malignancies [68].

Additionally, a new prognostic marker for patients with CRC has been described using the MALAT1, which functions as a predictive biomarker of metastasis in non-small cell lung cancer patients. MALAT1, CCAT1, and PANDAR are lncRNAs that are upregulated in the blood of CRC patients compared to healthy controls, which raises the possibility that they could serve as biomarkers for the prognosis of CRC [68].

By using statistical analysis and experimental validation, researchers recently discovered a ceRNA regulatory network, in which 23 differently expressed lncRNAs, 7 miRNAs, and 244 mRNAs function as regulatory axes connected to the development and prognosis of CRC tumors. IGF2-AS expression is positively linked with IGF2 expression in CRC patients. However, in the IGF2-AS/miR-150/IGF2 axis, over-expression of miRNA150 downregulates the expression of the lncRNA IGF2-AS, resulting in the overexpression of IGF2 [69]. PVT1 is another lncRNA linked to the etiology of CRC; its upregulation affects the downregulation of miR-16-5p, a key tumor suppressor in CRC. In a mouse xenograft model, the tumor volume has been seen to be significantly reduced by the deletion of PVT1 and miR-16-5p over-expression. Additionally, the etiology of CRC is intimately linked to the PVT1-miR-16-5p/VEGFA/VEGFR1/AKT axis: Upregulation of PVT1 results in the downregulation of miR-16-5p, which increases the levels of VEGFA and inhibits the activity of VEGFR1 and AKT [70]. A single nucleotide polymorphism at the PVT1 gene (8q24) is strongly related to an increased risk of developing CRC by genome-wide association studies. Four miRNAs are produced by the PVT1 locus: miRNAs 1204, 1205, 1206, and 1207-5p and -3p, some of which are crucial for the tumorigenic beginning of CRC and GC [71]. RP11-468E2.5 is one of the differentially expressed lncRNAs linked to the progression of CRC; nevertheless, compared to paired normal mucosa, its target genes STAT5A and STAT6 transcription factors are upregulated [72]. The effects of RP11-468E2.5 decrease on the activation of the JAK/STAT signaling pathway, which is driven by the induce of STAT5 and STAT6, elevating cell proliferation and inhibiting apoptosis in CRC, are also highlighted by the fact that silencing of RP11-468E2.5 results in an increase in the expressions of JAK2, STAT3, STAT5, STAT6, CCND1, and Bcl-2 [73].

Additionally connected to the development of CRC and carcinogenesis is the lncRNA MIR17HG. By sponging miR-375, MIR17HG promotes the expression of NF-B and RELA. In a positive feedback loop, RELA binds directly to the promoter region of MIR17HG to induce transcription. Among several miRNAs produced by MIR17HG, miR-17-5p increases the ability of CRC cells to move and invade by suppressing the expression of the tumor suppressor B-cell linker (BLNK). Additionally, MIR17HG upregulates PD-L1 expression, making it a potential therapeutic target [74].

The miR-574–5 sponged by the lncRNA MFI2-AS1 activates the production of MYC binding protein (MYCBP), boosting CRC cell proliferation and migration [75]. In both CRC cell lines and human patients, the lncRNA FEZF1 antisense RNA 1 (FEZF1-AS1) is also tightly linked to cell proliferation, migration, and invasion. Orthodenticle homeobox 1 (OTX1) expression levels are increased when FEZF1-AS1 expression levels are decreased [56], which indicates that the FEZF1-AS1/OTX1/EMT axis is involved in the formation of CRC. Additionally, through sponging miR-30a-5p, FEZF1-AS1 positively controls the expression of NT5E [76].

Through altering the miR-214-3p/LIVIN complex, the upregulation of the lncRNA DNAJC3 divergent transcript (DNAJC3-DT) is also closely linked to the advancement of CRC. MiRNA-214-3p is expressed at higher levels in CRC cell lines as a result of this deregulation, according to analysis of the downstream pathway using DNAJC3-DT silencing [77]. Therefore, the upregulation of miRNA-214-3p prevents LIVIN from being expressed as a protein and prevents the NF-B signaling pathway from being activated, both of which prevent the advancement of CRC. The lncRNA IGFL2 antisense RNA 1 (IGFL2-AS1) is a component of KRAS signaling, EMT, and angiogenesis. It has recently been demonstrated that it is associated with tumor cell proliferation and invasion and is overexpressed in colon adenocarcinoma tissue and CRC cell lines. Patients with colon adenocarcinoma have an independent tumor marker called IGFL2-AS1. Higher levels of IGFL2-AS1 are associated with a worse prognosis and may hasten the development of cancer [78].

The identification of important regulators by examining differentially expressed lncRNAs in GC and CRC tissues or cell lines would require further research and could offer fresh perspectives on lncRNA-based therapies in these cancers [79].

### LncRNAs as potential diagnostic and prognostic markers in GC and CRC

The expression of lncRNAs fluctuates in different disease states because lncRNAs play a role in controlling the development of GC and CRC. The aberrant expression of lncRNA in these cancer tissues, blood, or plasma may serve as a novel tumor diagnosis marker. Furthermore, the estimate or forecast of a disease's future course and the likelihood of recovery or survival from illnesses, such as cancer, is known as the prognosis. The mechanism of GC and CRC and their therapy need to be better understood in order to predict survival, which is a difficult task. The lack of early, reliable prognostic signs is one of the main causes of the poor prognosis for GC and CRC [80].

The clinical stage of CRC patients was associated with an increase in the expression level of the lncRNA UICLM in tumor tissues. In addition, individuals with liver metastases had considerably higher levels of UICLM expression in their tumor tissues than patients without liver metastases [81]. Lnc34a expression was shown to be significantly higher in advanced CRC specimens compared to earlier tumor specimens, as reported, indicating that Lnc34a expression may be a possible biomarker for advanced stages. MIR17HG may be one of the markers for the early diagnosis of CRC to identify adenoma from adenocarcinoma because it was found that the expression of MIR17HG in colorectal adenocarcinoma was higher than that in normal tissues and adenomas [81]. PVT1 at CpG site (CG23898497) was more methylated in CRC patients than in healthy individuals. Consequently, it is regarded as a prognostic and diagnostic indicator of CRC. To assess the relationship between FLANC expression levels and CRC, performed in situ hybridization (ISH) using tissue microarrays and found that: overall, FLANC expression was significantly higher in adenocarcinoma and metastatic lesions than in normal, benign polyp and inflammatory colon tissues [82, 83]. Nevertheless, upregulation of FLANC may occur in malignant epithelial cells, and therefore, FLANC may be a sensitive indicator for early diagnosis of CRC and may specifically distinguish tumor from benign disease [84]. High MALAT1 expression may be employed as a marker of advanced CRC and recurrence because it was directly correlated with the CRC pathology stage and tumor recurrence. miR00HG's function is mediated by let-7a-2-3p, miR-125b-5p, and miR-125b-1-3p expression, all of which are encoded by intron-3, suggesting that miR100HG may be one of the markers for predicting CRC tumor stage [85]. A study found that the expression levels of miR100HG differed significantly in stage I, II, and III/IV CRC tissues [86]. Additionally, with considerably different results, RP11 was found in normal and CRC tissues at various stages, and it has good specificity in both CRC cell lines and clinical tissues, suggesting that it could be one of the novel markers for early diagnosis. A more accurate prognosis of CRC was obtained when GLCC1 expression and TNM staging were combined [87]. Different stage N CRC patients express KRT7-AS in dramatically different ways, suggesting that KRT7-AS may be utilized to predict the lymph node metastasis of CRC. NEAT1 in blood could be a crucial component of early CRC screening due to its high sensitivity and specificity in identifying colorectal cancer patients from healthy control patients, both of which were 83.3%. Various CRC stages can be predicted by H19, and CCAT2 and EVADR can predict CRC metastasis [88].

CCAT1 has been identified as a potential diagnostic and prognostic marker in CRC. It is linked to tumor differentiation, lymph node metastasis, distant metastasis vascular invasion, overall survival, and recurrence-free survival [89]. In addition to MALAT1, CCAT1 and PANDAR in the blood can serve as probable factors for CRC prognosis [90]. Likewise, BLACAT1, and CRNDE in blood are used as diagnostic markers based on minimally invasive liquid biopsy [91, 92].

RP11-296E3.2 and LEF1-AS1 are recognized as diagnostic and prognostic markers for CRC metastasis [93]. Upregulation of lncRNA TPTEP1, ZDHHC8P1, and ALMS1-IT1 is also a prognostic factor for overall survival in CRC and is significantly associated with CRC advancement [94–96].

The upregulation of lnc-HSD17B11-1:1, LINC02257, AC156455.1, and AC104532.2 in CRC acts as a prognostic and diagnostic marker. Moreover, suppression of LINC02257 significantly inhibits cell proliferation [97–99]. MEG3's decreased expression serves as both a diagnostic and prognostic indicator for patients with CRC [100]. Increased expression of lncRNA SUMO1P3 as an oncogene and prognostic marker represses proliferation and apoptosis in CRC by inhibiting CPEB3 [101]. Furthermore, lncRNA-CCHE1, identified as an oncogene and prognostic factor, is linked to characteristics such as tumor size, advanced stage, metastasis lymph node, and vascular aggression [102].

Furthermore, the expression level of ADAMTS9-AS1 and LINC01410 is significantly increased in CRC, which correlates with TNM stage, lymph node metastasis, and poor survival and prognosis [103, 104].

Nevertheless, the high expression of LBX2 antisense RNA 1 (LBX2‑AS1) is a sensitive prognostic and diagnostic factor for CRC. Its inhibition stops the growth, proliferation, migration, and aggression of the tumor [105]. Upregulation of LUNAR1 in CRC is linked to antagonistic tumor characteristics, reduced overall survival and disease-free survival (DFS). Conversely, suppressing the prognostic marker LUNAR1 hampers tumor expansion, migration, aggression, and growth [106].

A bioinformatic review reported several lncRNAs including LINC00543, PTPRD-AS1, AP003555.1, AC009237.14, and AL109615.3 as prognostic markers for CRC [107]. Upregulation of TMPO-AS1 is notably associated with lymph nodes and distant metastasis and like HOTTIP are considered as a prognosis of CRC [108, 109]. Alternatively, increased expression of LINC01314 is associated with vascular metastasis and also plays a prognostic and diagnostic role in CRC [110]. LINC01106 controls the growth and programmed cell death of CRC cells by modulating the STAT3/Bcl-2 signaling pathway. This suggests that LINC01106 has the potential to be a useful marker for diagnosing and prognosis of CRC [111].

Likewise, MIR31HG and WASIR2 demonstrated particular consequences as prognostic indicators for stage II CRC [112]. Decreased expression of ANRIL and increased expression of BANCR have also been confirmed as prognostic and early diagnosis of CRC [113]. Inhibiting epidermal growth factor receptor (EGFR) signaling through suppressing the prognostic factor linc-ROR leads to a decrease in aggression, proliferation, and migration in CRC [114].

Plasma HOTAIR has sensitivity and specificity for the diagnosis of GC. B3GALT5-AS1 in plasma can also function as a diagnostic biomarker to separate GC patients from healthy controls. In comparison with healthy controls, GC patients have plasma levels of H19 that are considerably more significant [115].

Multiple lncRNA combinations also demonstrate improved diagnostic biomarker values for GC. For instance, GC patients have considerably higher plasma levels of the lncRNAs AK001058, INHBA-AS1, MIR4435-2HG, and CEBPA-AS1 than healthy controls. These plasma lncRNAs work better together than individually as diagnostic biomarkers of GC.

In comparison with healthy controls, GC patients had considerably higher expression levels of the lncRNAs PANDAR, FOXD2-AS1, and SMARCC2 plasma. GC patients have higher plasma levels of the lncRNAs AFAP1-AS1 and FEZF1-AS1 than healthy individuals [116].

When compared to nearby control tissues, GC tissues express the lncRNA TUG1 more, and this expression is positively correlated with cancer invasion and stage. A unique predictor of the prognosis of GC may be higher TUG1 expression, which is linked to a worse prognosis. Many GC cell lines have greater levels of the lncRNA SNHG3 expression. Results from GC cells in both in vitro and in vivo settings have demonstrated that SNHG3 can encourage cell growth and metastasis. TUG1 overexpression is connected to poor clinical outcomes [117]. In GC patients, the lncRNA UCA1 is increased, which encourages GC cell growth and migration. A worse overall survival is linked to increased UCA1 expression. All of these investigations show a connection between dysregulated lncRNAs and GC patients' survival. Additionally, the survival of GC patients can be predicted by differences in a few lncRNAs. H19, CRNDE, HOTAIR, and MALAT1 are lncRNAs that have prognostic significance in CRC [118]. The expression of H19 was revealed to be an independent predictor of OS and DFS, and it was found to be connected with tumor differentiation and advanced TNM stage. The negative prognosis in CRC could be predicted by H19 lncRNA overexpression, according to several research. Due to its high tissue and serum exosome levels, which are strongly linked with tumor size, lymph node status, and distant metastasis, CRNDE-h has the potential to be a useful early diagnostic biomarker for CRC. Furthermore, elevated exosomal CRNDE-h levels have been shown to be a poor predictor of OS in CRC patients [119].

In addition to previously mentioned lncRNAs such as HOTAIR, PVT1, H19, and UCA1, LINC00152 has also been identified as a probable diagnostic and prognostic marker for GC [120].

The level of linc-ROR and SSTR5-AS1 expression in GC tissues is notably reduced when compared to the surrounding non-tumorous tissues. Moreover, GC patients exhibiting elevated expression levels of linc-ROR and SSTR5-AS1 demonstrated a significantly improved overall survival rate in comparison to patients with lower expression levels [121, 122]. Several lncRNAs regulation including HAGLR, LINC00392, LINC01729, LINC01094, U95743.1, AP000695.1, AC005332.1, AC009812.4, AC007785.3, AC079385.3, AC016394.2, AC023511.1, AC147067.2, and AL590705.3 have been identified as prognostic markers for GC. Furthermore, the lncRNA AC007785.3 has been associated with GC metastasis [123–125]. The expression level of lncRNA SNHG5 was found to be significantly reduced in preoperative GC patients and individuals with a poor prognosis. This decrease in SNHG5 expression highlights its potential as a valuable diagnostic tool for predicting prognosis in GC [126, 127]. Furthermore, elevated expression of SNHG17 and lncRNA MVIH in GC tissues, when compared to adjacent normal tissues, has been linked to advanced stage, lymph node and distant metastasis, and poor overall survival outcomes [128, 129].

Tumor tissues exhibited decreased expression of prognostic markers LINC02410, AC012317.2, and AC141273.1, whereas AC019117.2 and LINC00941 exhibited elevated expression in tumor tissues compared to normal tissues. Moreover, AC019117.2 and LINC00941 demonstrated a significant correlation with tumor stages of GC [130]. Besides, PVT1 identified as an unfavorable prognostic biomarker, exhibits a significant association with factors such as gender, aggression, poorer overall survival, and more destructive DFS in GC [131]. Moreover, the combination of PVT1 expression level with carbohydrate antigen 19–9 (CA19-9) has the potential to serve as a more effective diagnostic marker in GC [132].

Other prognostic elements of GC include MIAT exosomal lncRNA, which has high expression in gastric adenomas that are prone to GC. On the other hand, after treatment, its expression reduces extremely [133]. GC patients with higher expression levels of HEIH (Hepatocellular Carcinoma Upregulated EZH2-Associated Long Noncoding RNA) exhibited a poorer prognosis in comparison to those with lower HEIH expression (134). Aberrant expression of CCDC144NL-AS1 is also a prognostic biomarker in gastric cancer, like some other cancers [135]. Additionally, clinical data have revealed a correlation between age and clinical pathological stage with GC prognosis [136].

LINC01644, LINC01697, and CERS6-AS1 are overexpressed in GC cells and play a significant role in prognosis. Inhibition of LINC01644, LINC01697 and CERS6-AS1 repressed GC cell expansion [137, 138]. As well, the downregulation of LINC00086 and neighboring enhancer of FOXA2 (NEF) showed high sensitivity and specificity in the diagnosis of GC with downregulation [139, 140]. Upregulation of LINC00205 is associated with an unfavorable prognosis and shows potential as a plasma identification for tumor diagnosis [141].

AP000695.2 aberrant expression with poor survival has been informed as a prognostic and diagnostic biomarker in GC [142]. The upregulation of LINC00365, acting as a tumor suppressor, has the potential to serve as a prognostic marker in GC. It suppresses GC cell viability by inhibiting expansion rather than stimulating apoptosis [143]. Additionally, LINC01235 functions as a prognostic biomarker and facilitates the metastasis of GC cells by inducing EMT [144].

LINC01279 exhibits elevated expression levels in both GC tissues and serum and is associated with tumor aggression. Serum LINC01279 serves as a superior prognostic factor for advanced cancer [145]. The elevated expression of RP11-357H14.17 and FOXP4-AS1 in GC is linked to reduced overall survival and an unfavorable prognosis. RP11-357H14.17 regulation is also linked with clinical characteristics such as tumor size, differentiation, and metastasis [146, 147]. The decreased expression of LINC01939 demonstrated a relation with metastasis and poor prognosis and overall survival in GC [148].

Based on the above findings, lncRNAs play important roles in gene regulation and are increasingly being recognized as potential diagnostic and prognostic biomarkers for cancer. Table 1 summarizes the clinical implications of various lncRNAs which differentially expressed in CRC and GC compared to normal samples, as well as lncRNAs that are associated with tumor stage, lymph node metastasis, survival, and other clinical outcomes.

**Table 1:** A summary of various lncRNAs and their roles in the diagnosis and prognosis of GC and CRC

Cancer type	LncRNAs		References
	Diagnostic role	Prognostic role	
CRC	MIR17HG, PVT1, FLANC, MALAT1, miR100HG, RP11, NEAT1, H19, CCAT1, PANDAR, BLACAT1, CRNDE, RP11-296E3.2, LEF1-AS1, LINC02257, MEG3, SUMO1P3, lncRNA-CCHE1, ADAMTS9-AS1, LINC01410, LBX2-AS1	UICLM, Lnc34a, PVT1, FLANC, MALAT1, miR100HG, GLCC1, KRT7-AS, CCAT2, EVADR, CCAT1, PANDAR, RP11-296E3.2, LEF1-AS1, TPTEP1, ZDHHC8P1, ALMS1-IT1, LINC02257, MEG3, SUMO1P3, lncRNA-CCHE1, ADAMTS9-AS1, LINC01410, LBX2-AS1, LUNAR1, LINC00543, PTPRD-AS1	[81–114]
GC	HOTAIR, B3GALT5-AS1, H19, AK001058, INHBA-AS1, MIR4435-2HG, CEBPA-AS1, TUG1, SNHG3, UCA1, linc-ROR, SSTR5-AS1, MVIH, MIAT, LINC00086, NEF, LINC00205, LINC01279	PANDAR, FOXD2-AS1, SMARCC2, AFAP1-AS1, FEZF1-AS1, TUG1, SNHG3, UCA1, H19, CRNDE, MALAT1, LINC00152, linc-ROR, SSTR5-AS1, HAGLR, LINC00392, LINC01729, LINC01094, U95743.1, AP000695.1, AC005332.1, AC009812.4, AC007785.3, AC079385.3, AC016394.2, AC023511.1, AC147067.2, AL590705.3, SNHG5, HEIH, CCDC144NL-AS1, LINC01644, LINC01697, CERS6-AS1, LINC00205, AP000695.2, LINC00365, LINC01235, LINC01279	[115–148]

### Most important signaling pathways in gastric and colorectal Carcinogenesis

To sustain homeostasis, pro-growth, and anti-apoptotic signals must coexist in a delicate equilibrium. This process involves a number of signaling pathways, and many of these pathways' constituents are frequently altered by cancer, resulting in dysregulation. We may get new insights into GC and CRC development by better understanding how lncRNAs regulate signaling pathways [149] (Table 2).

**Table 2:** lncRNA's functions and related signaling pathways in GC and CRC

Cancer	LncRNA	Regulation	Signaling pathways	Function	References
CRC	PINT	Down`	PRC2	Inhibits proliferation	[186]
	MALAT1	Up	miR-129-5p, HMGB1, miR-663a	Improve proliferation, migration and invasion	[187]
	Loc285194	Down	miR-211	Inhibits proliferation	[188]
	Linc02418	Up	miR-34b-5p, Bcl-2	Stimulates growth, mobility, invasion, apoptosis inhibition	[189]
	Linc00261	Down	β-catenin, Wnt	Inhibits proliferation, apoptosis, invasion, migration, and drug resistance	[190]
	Linc01567	Up	miR-93	Stimulates proliferation, invasion, migration	[191]
	H19	Up	miR138, HMGA1, miR-675-5p	Improve invasion, migration, and drug resistance	[119]
	HOTAIR	Up	E-cadherin, vimentin, MMP-9	Increases migration, invasion	[192]
	GSEC	Up	DHX36	Elevates migration	[193]
	FAM83H-AS1	Up	TGF-β signaling	Elevates tumorigenesis	[194]
	HNF1A-AS1	Up	miR-34a/p53	Enhances proliferation, migration, invasion	[195]
	HULC	Up	miR-613, RTKN, vimentin, N-cadherin, E-cadherin	Elevates proliferation, migration, invasion	[196]
	FAL1	Up	STAT3, TGF-β1, Bcl-2, p65, PCNA	Enhances proliferation, invasion, inhibits apoptosis	[197]
	DACOR1	Down	Cystathionine β-synthase	Inhibits proliferation, increases DNA methylation	[198]
	CCAT1	Up	c-Myc	Promotes proliferation, invasion, drug resistance	[199]
	CCAT2	Up	miR-145, WNT	Enhances growth, metastasis	[199]
	CASC15	Up	miR-4310, LGR5, Wnt/β-catenin	Promotes proliferation, migration, invasion	[200]
	CASC19	Up	N/A	Increases migration	[201]
	ATB	Up	E-cadherin, ZO-1, ZEB1, N-cadherin	Enhances invasion, induces EMT	[202]
	PVT1	Up	miR-26b, miR-30d-5p/RUNX2	Elevates proliferation, migration, invasion	[203]
	ROR	Up	miR-145	Enhances proliferation, migration, invasion	[204]
	TUG1	Up	miR-26a-5p, MMP-14, VEGF, MAPK, Hsp27	Promotes proliferation, migration, invasion, EMT, inhibits apoptosis	[205]
	UPAT	Up	UHRF1	Elevates survival	[206]
	XIST	Up	miR-34a, Wnt, β-catenin cyclin D1, c-Myc, MMP-7	Promotes proliferation	[207]
	ZEB1-AS1	Up	miR-455-3p, PAK2	Enhances proliferation, migration, invasion	[208]
	ZFAS1	Up	ZEB1, E-cadherin, ZO-1, vimentin, N-cadherin	Increases proliferation, invasion, EMT, inhibits apoptosis,	[209]
	SNHG1	Up	Wnt/β-catenin, c-Myc, cyclin D	Increases proliferation, invasion, migration, inhibits apoptosis	[210]
	SNHG7	Up	miR-193b, K-ras/ERK/cyclinD1	Increases proliferation, inhibits apoptosis	[211]
	SNHG15	Up	Slug	Enhances proliferation, migration	[212]
	SNHG17	Up	miR-375, CBX3	Elevates proliferation, migration, invasion	[213]
	Linc01106	Up	miR-449b-5p, Gli	Confers proliferation, migration, stemness	[214]
	Linc01234	Up	miR-642a-5p, SHMT2	Promotes proliferation	[215]
	Linc00657	Down	CAPN7, PI3K/Akt	Inhibits proliferation, invasion, induces apoptosis	[216]
	Linc01578	Up	NF-κB, YY1	Enhances metastasis	[217]
	CYTOR	Up	β-catenin/TCF complex	Promotes migration, invasion, EMT	[218]
	BC200	Up	STAT3, β-catenin	Increases proliferation, invasion, EMT, apoptosis inhibition, cell cycle regulation	[219]
	LincDUSP	Up	ATR, p53, E2F, c-Myc	Promotes proliferation, stem cells, modulates DNA damage response and cell cycle, inhibits apoptosis	[220]
	USP2-AS1	Up	Phosph-YAP	Enhances proliferation, metastasis	[221]
	LINC00152	Up	MAPK	LINC00152 suppression significantly reduces the expression of p-ERK-1/2, p-MEK1/2, and c-fos without involving the ERK-1/2 and MEK1/2 expressions. SA treatment changes the function of LINC00152	[222]
	PICART1	Down	MAPK	The upregulation of PICART1 reduces the pERK expression, whereas PICART1 silencing reverses the expression of p-ERK	[223]
	AOC4P	Up	MAPK	The suppression of AOC4P reduces the expression of ERK1, JNK, and p38	[224]
GC	linc00483	Up	MAPK	The suppression of Linc00483 reduces the expression of c-Jun without affecting the p-Jnk, p53, and p-p38 expression	[225]
	CRNDE	Up	PI3K/AKT	The repression of CRNDE significantly downregulates pPI3K and p-Akt expression	[226]
	LINC01559	Up	PI3K/AKT	The suppression of LINC01559 reduces the expression of PGK1, p-PI3K, p-AKT, and mTOR. IGF-1 treatment (PI3K activator) significantly reverses the LINC01559 knockdown induced phosphorylation of PI3K, AKT, and mTOR	[227]
	PICART1	Down	PI3K/AKT	The upregulation of PICART1 reduces p-AKT expression, whereas PICART1 inhibition increases p-AKT expression	[223]
	LINC01419	UP	PI3K/AKT	The suppression of LINC01419 in GC cells reduces the expression of p-AKT1 and p-mTOR but does not affect their total levels	[228]
	LOC101928316	Down	PI3K/AKT	LOC101928316 upregulation decreases the expression levels of PI3K, p-AKT, mTOR, and p-mTOR. However, the knockdown of LOC101928316 upregulates AKT3, mTOR, and p-mTOR expression and suppresses PTEN expression	[229]
	PCAT18	Down	PI3K/AKT	PCAT18 inhibits the PTEN/PI3K/AKT signaling pathway by sponging miR-107, leading to the inhibition of GC progression	[230]
	OGFRP1	UP	PI3K/AKT	The silence of OGFRP1 downregulates the expression of p-AKT, leading to the suppression of cell-cycle progression and induction of apoptosis	[231]
	ADAMTS9-AS2	Down	PI3K/AKT	The inhibition of ADAMTS9-AS2 increases the expression of p-PI3K and p-AKT. The administration of the PI3K inhibitor LY294002 reverses the negative effect of ADAMTS9-AS2	[232]
	FOXD1-AS1	UP	PI3K/AKT	FOXD1-AS1 activates the PI3K/AKT/mTOR pathway via the upregulation of PIK3CA, leading to an aggravation of GC progression and chemoresistance	[233]
	TMPO-AS1	UP	PI3K/AKT	The silence of TMPO-AS1 inhibits the PI3K/ AKT/mTOR signaling pathway by downregulating the expression of BRCC3 via releasing miR-126-5p	[234]
	XLOC_006753	UP	PI3K/AKT	X LOC_006753 knockdown reduces the expression of PI3K, p-AKT (Thr308), p-AKT (Ser473), and p-mTOR (Ser2448), causing to the enhance of MDR	[235]
	MIR4435-2HG	UP	Wnt/ β-catenin	The suppression of MIR4435-2HG reduces β-catenin expression in GC xenografts and inhibits the trans-activating activity of β-catenin	[236]
	GASL1	DOWN	Wnt/ β-catenin	The upregulation of GASL1 reduces β-catenin expression in GC cells, while GASL1 knockdown increases β-catenin expression. The administration of a Wnt agonist reduces the negative function of GASL1 on GC cell	[237]
	LINC01314	DOWN	Wnt/ β-catenin	The overexpression of LINC01314 downregulates the expression of Wnt-1, β -catenin, cyclin D1, and N-cadherin, while it upregulates E-cadherin expression	[238]
	FAM83H-AS1	UP	Wnt/ β-catenin	Silencing FAM83H-AS1 decreases the expression of β-catenin	[239]
	ZEB2-AS1	UP	Wnt/ β-catenin	ZEB2-AS1 activates the Wnt/ β-catenin signaling pathway by upregulating ZEB2 expression in GC cell	[240]
	LINC00665	UP	Wnt/ β-catenin	LINC00665 knockdown decreases the expression of β-catenin and cyclin D1 in GC cells, whereas it increases GSK-3b expression	[241]
	TOB1-AS1	DOWN	Wnt/ β-catenin	The knockdown of TOB1-AS1 increases the expression of β-catenin, c-Myc, cyclin D1, and N-cadherin	[241]
	lincRNA-p21	DOWN	Wnt/ β-catenin	The upregulation of lincRNA-p21 reduces the expression of β-catenin and c-Myc	[242]
	GATA6-AS1	DOWN	Wnt/ β-catenin	The upregulation of GATA6-AS1 downregulates β-catenin levels and decreases intranuclear β -catenin expression. In GATA6-AS1-silencedGC cells treated with LiCl, β-catenin expression is upregulated	[243]
	HCG11	UP	Wnt/ β-catenin	The suppression of HCG11 inhibits the proliferation of GC cells by inhibiting the activity of the Wnt signaling pathway. The administration of LiCl the effect of HCG11 silence on the proliferation	[244]
	LINC01133	DOWN	Wnt/ β-catenin	LINC01133 suppresses the nuclear accumulation of β-catenin in GC cells by sponging miR-106a-3p and promoting APC expression	[245]
	LINC01503	UP	Wnt/ β-catenin	Silencing LINC01503 in GC cells reduces the expression of β-catenin, cyclin D1, and c-Myc, whereas LINC01503 upregulation reverses their expression	[246]
	LINC01225	UP	Wnt/ β-catenin	LINC01225 knockdown reduces the expression ofWnt1and β-catenin in GC cells, whereas it does not affect the expression or Ser9 phosphorylation of GSK-3b	[247]
	BANCR	UP	NF-kB	BANCR silence decreases the expression of NFkB1 (P50/105) and inhibits the activity of NF-kB1 30 UTR	[248]
	LINC01410	N/A	NF-kB	The upregulation of LINC01410 enhances the nuclear signals of NF-kBp65 and the expression of p-IKK-b, p-IkBa, and c-FLIP in GC cells, whereas LINC01410 reverses their expression	[249]
	KRT19P3	DOWN	NF-kB	KRT19P3 inactivates the NF-kB signaling pathway by promoting the degradation of IkBa induced by COPS7A suppression	[250]
	ANRIL	N/A	NF-kB	The inhibition of ANRIL in GC cells reduces the proteinlevelsofp65in the nucleus and the mRNA levels of NF-kB target genes	[251]
	ASB16-AS1	UP	NF-kB	ASB16-AS1 activates the NF-kB signaling pathway by upregulating TRIM37 expression	[252]
	NALT1	UP	NOTCH	NALT1 suppression reduces the expression of NOTCH1, NICD, and the downstream target genes of the notch signaling pathway, consisting of HES1 and HES5	[253]
	MIR22HG	DOWN	NOTCH	The suppression of MIR22HG enhances the expression of HEY1 and nucleus NOTCH2	[254]
	EGOT	UP	Hedgehog	The inhibition of EGOT reduces the expression of Shh, SUFU, and Gli1 at both the transcription and protein levels	[255]
	HOXD-AS1	UP	STAT3	The suppression HOXD-AS1 downregulates the expression of p-JAK2 and p-STAT3	[256]
	SNHG16	UP	STAT3	The knockdown of SNHG16 in GC cells reduces the expression of JAK2 and p-STAT3 by sponging miR-135a	[257]
	NEAT1	UP	STAT3	Silencing NEAT1 decreases STAT3 expression by sponging miR-506	[258]
	PVT1	UP	STAT3	PVT1 upregulation enhances the accumulation of p-STAT3 in the nucleus by inhibiting its ubiquitin-dependent degradation and increases the transcriptional activity of STAT3	[259]
	CTC-497E21.4	UP	RhoA	The suppression of CTC-497E21.4 regulates the expression of total and active RhoA, CDC42, and Rac1 in GC cells	[260]
	NORAD	UP	RhoA	Silencing NORAD in GC cells decreases the expression of RhoA and ROCK1	[261]
	HOTAIR	UP	RhoA	The upregulation of HOTAIR increases the expression of CXCR4, RhoGEF, PI3K, ROCK, PAK, and PKN	(262)
		UP	STAT3	Silencing HOTAIR reduces the expression of STAT3 and cyclin D1	

### Wnt/ β-catenin signaling pathway

Proliferation, stem cell maintenance, and homeostasis are some of the basic functions of the normal gastric mucosa that are regulated by the Wnt/ β-catenin signaling pathway on the other way, is one of the evolutionarily conserved pathways frequently observed in CRC. The Wnt pathway is accountable for cell growth and multiplication in adult tissues as well as during embryonic development. Depending on whether β-catenin in is involved, Wnt signaling is either canonical or noncanonical [150, 151]. The accumulation of β-catenin in the nucleus is linked to a more aggressive form of the disease Several GC patients have abnormal activation of the Wnt/β-catenin signaling pathway, which affects several facets of GC progression. By controlling the expression of essential elements in the Wnt/ β-catenin signaling pathway, some lncRNAs, including GASL1, HCG11, LINC00665, LINC01503, lincRNA-p21, LINC01314, LINC01225, and FAM83H-AS1, have been demonstrated to have an impact on the carcinogenesis of GC. Other lncRNAs have also been shown to indirectly modify the Wnt/β-catenin signaling pathway by sponging miRNAs as ceRNAs, including CASC15, SNHG11, and NCK1-AS1 [152]. Additionally, it has been discovered that the lncRNA HOXC-AS1 can inhibit eIF4AIII, leading to an increase in the expression of β-catenin [152]. By preventing Frizzled 4 expression, GATA6-AS1 has been found to deactivate the Wnt/-catenin signaling pathway. Together, these results show that lncRNAs in GC have a major effect on the Wnt/β-catenin signaling pathway [153].

Wnt activation also adversely correlates with T cell and B cell infiltration in a teratoma model, which is typically linked to a worse clinical outcome. Wnt/ β-catenin overexpression is linked to the dysregulation of numerous ncRNAs in CRC, which has a subsequent clinical impact. Scilicet, CCAL is an important factor in the development of CRC tumors. Its high expression is associated with lower therapeutic response and overall survival [154]. This is because AP-2 is activated by the Wnt/ β-catenin pathway, which is downregulated by CCAL. Another lncRNA, HCG18, which is elevated in CRC patient samples as well as in CRC cell lines and whose expression is negatively linked with miR-1271 levels, also activates this signaling pathway [155]. MiR-1271 can be sponged by HCG18, and blocking it prevents tumor development and invasion. TDRKH-AS1 overexpression in CRC also activates the Wnt/β-catenin pathway. MiR-29b-3p is a target of H19, which is frequently elevated in CRC. H19 promotes the EMT by upregulating its target protein PGRN via sponging miR-29b-3p, which in turn upregulates the downstream Wnt signaling [156].

### MAPK signaling pathway

The MAPK signaling pathway is well known to be tightly linked to essential biological processes such as cell proliferation, apoptosis, migration, invasion, and senescence. Numerous malignancies, including GC and CRC, have been found to have dysregulated MAPK signaling. Silencing lncRNA LINC00152-1 dramatically reduces the expression of p-ERK-1/2 and p-MEK-1/2 in GC cells while having no effect on the expression of total ERK-1/2 and MEK-1/2, demonstrating the significance of LINC00152-1 in promoting the MAPK signaling pathway [30]. Stavrosporine aglycone, an activator of the ERK/MAPK signaling pathway, counteracts LINC00152-1's impact on the cellular processes of GC cells. Additionally, overexpressing the lncRNA CASC2 in the GC BGC-823 cell line drastically reduces the expression of p-ERK1/2 and p-JNK, whereas p-p38 expression is unaffected. Another study found that downregulating lncRNA AK025387 reverses the expression of Raf-1, MEK2, and ERK in GC cells while upregulating these proteins' expression in MKN45 and SGC7901 GC cells [157]. Additionally, it has been suggested that several lncRNAs, including AOC4P, LINC00483, and BCAR4, contribute to the advancement of GC through direct or indirect modulation of the MAPK signaling pathway. Besides, PVT1 on miR-152-3p's target E2F3 activates MAPK8 by sponging the signal directly to MAPK8. E2F3's target, lncRNA on miR-152-3p, sends the signal to MAPK8 directly in CRC [158].

### Sonic Hedgehog (Shh) signaling pathway

Another evolutionarily conserved system that is essential for the preservation of stem-like characteristics and appropriate differentiation is the hedgehog signaling pathway. The Smo G protein-coupled receptor is inhibited by the Shh protein. The Gli1 gene, among others, begins to be transcribed as a result of its action, creating a positive feedback loop. It also causes the Gli1 transcription factor to go into the nucleus [159]. Wnt, EGFR, and Notch pathways, in particular, as well as other significant pathways involved in proliferation and differentiation, interact with hedgehog signaling. Hedgehog signaling in CRC is triggered by lncRNA-cCSC1. The stem cells of colorectal cancer are strongly expressed [160]. Depletion of lncRNA-cCSC1 decreases the ability of CRC stem cells to self-renew and increases sensitivity to 5-FU, a drug frequently used to treat CRC. This makes lncRNA-cCSC1 a possible therapeutic target. Correspondingly, in GC, Shh, SUFU, and Gli1 expression is both downregulated when lncRNA EGOT is silenced, showing that EGOT has a deleterious effect on the Hedgehog signaling pathway [161].

### PI3K/AKT signaling pathway

It has been demonstrated that the PI3K/AKT signaling pathway controls the proliferation, metastasis, and treatment resistance of GC cells. The deregulation of the PI3K/AKT signaling pathway and the development of GC are tightly connected. Additionally, it has been noted that lncRNAs play a crucial role in controlling the PI3K/AKT signaling pathway [162]. In one example, overexpression of the long noncoding RNA (lncRNA) LOC101928316 drastically reduces the expression of PI3K, p-AKT, mTOR, and p-mTOR in the human GC cell line SGC-7901. This finding suggests that LOC101928316 is implicated in the progression of GC by inhibiting the PI3K/AKT signaling pathway. In GC samples and MDR GC cell lines, lncRNA XLOC_006753 is considerably elevated [163]. In MDR GC cells, XLOC_006753 knockdown reduces the expression of PI3K, p-AKT, and p-AKT. The modulation of the PI3K/AKT signaling pathway in GC has also been linked to various other lncRNAs, including OGFRP1, TMPO-AS1, and FOXD1-AS1. All of these results point to the possibility that lncRNAs influence the PI3K/AKT signaling pathway in either a pro- or anti-GC manner [162].

### STAT3 signaling mechanism

Given that STAT3 has been linked to the development of chemotherapy resistance and the proliferation, angiogenesis, and invasion of cancer cells, it is a potential therapeutic target in GC. LncRNAs are essential upstream regulators of the STAT3 signaling pathway in GC, according to mounting data. PVT1 directly interacts with active p-STAT3 protein to increase its stability, causing p-STAT3 to accumulate in GC cells' nuclei and activating the STAT3 signaling pathway. By directing STAT3 to the promoter of the STAT3 target gene PDK1, AC093818.1 aids in the transcriptional activation of the gene [164]. By improving the stability of the IL-11 mRNA, the lncRNA OLC8 encourages the STAT3 signaling pathway. It has been discovered that several lncRNAs, such as HOTAIR, SNHG16, NEAT1, and GACAT3, can indirectly control the STAT3 signaling pathway by sponging miRNAs as ceRNAs. Additionally, some lncRNAs, including HOXD-AS1, TRPM2-AS, and LINC00691, have been found to control the expression of crucial STAT3 signaling pathway constituents [165].

### PI3K/AKT signaling pathway

PI3K/AKT signaling pathway controls the proliferation, metastasis, and treatment resistance of GC cells. The deregulation of the PI3K/AKT signaling pathway and the development of GC are tightly connected. Additionally, it has been noted that lncRNAs play a crucial role in controlling the PI3K/AKT signaling pathway. overexpression of LOC101928316 drastically reduces the expression of PI3K, p-AKT, mTOR, and p-mTOR in the human GC cell line SGC-7901 [162]. It is implicated in the progression of GC by inhibiting the PI3K/AKT signaling pathway. In GC samples and the multidrug-resistant GC cell lines, lncRNA XLOC_006753 is considerably elevated, according to another study. In MDR GC cells, XLOC_006753 knockdown reduces the expression of PI3K, p-AKT, and p-AKT. The modulation of the PI3K/AKT signaling pathway in GC has also been linked to various other lncRNAs, including OGFRP1, TMPO-AS1, and FOXD1-AS1. All of these results point to the possibility that lncRNAs influence the PI3K/AKT signaling pathway in either a pro- or anti-GC manner [166].

### C-MYC signaling pathway

The Wnt route, MAPK signaling stimulated by growth and survival factors, Jak/STAT signaling, and the TGF-signaling pathway are only a few of the signaling pathways that control the activity of the transcription factor C-MYC. In some human malignancies, C-MYC is elevated through a variety of mechanisms including amplification, mutation, and protein stabilization, which results in chronic proliferative and antiapoptotic signaling. C-MYC overexpression was demonstrated to cause innate cell infiltration and increased production of checkpoint inhibitors by tumor cells in a liver cancer model. These events collectively promoted tumor cell angiogenesis, chemoresistance, and proliferation [167].

In CRC, C-MYC activation was induced by lncCMPK2. Far upstream element binding protein 3 (FUBP3) is bound by lncCMPK2, which is localized in the nucleus. In such a complex, it directs FUBP3 to the FUSE (far upstream element) of c-Myc, where it then activates transcription. Likewise, lncCMPK2 is increased in comparison to normal epithelium, and in patient samples, this overexpression is correlated with tumor size, local lymph node metastases, and TNM staging. Additionally, it is known that GLCC1 speeds up CRC carcinogenesis by stabilizing C-MYC and preventing its ubiquitination. On the other hand, increased MIR31HG (lncRNA precursor molecule of miR31-3p and miR31-5p) expression results in the downregulation of C-MYC targets and TNF-/NF-B, TGF-, IFN-, and IFN- signaling gene signatures, as well as the subsequent inadequate differentiation [168]. Additionally, MIR31HG is a prognostic marker and a potential stratification factor for CRC that can be used regardless of whether cytotoxic T-lymphocytes or fibroblasts have invaded the tumor. MiR-31-3p causes cetuximab resistance in metastatic CRC, whereas miR-31-5p itself is a well-known poor prognostic marker in CRC. There is a protooncogene that is associated with C-MYC called MYC binding protein (MYCBP). MiR-495-3p is a negative regulator of it. Upregulation of lncRNA in CRC MiR-495-3p sponging by LUNAR1 reactivates MYCBP. In CRC cells, LUNAR1 knockdown restricts proliferation, migration, and progression and encourages apoptosis [169].

### Drug resistance is regulated by lncRNAs

For cancer therapy to be more effective, overcoming drug resistance is a significant obstacle, and lncRNAs have been linked to the emergence of drug resistance. The expression of five lincRNAs, including LINC00973, LINC00941, CASC19, CCAT1, and BCAR4, is consistently altered in HT-29 and HCT-116 cells after treatments with 5-FU, oxaliplatin (OXA), and irinotecan both in vitro and in vivo [170]. The most markedly elevated of these five lincRNAs in colon cancer cells treated with 5FU, oxaliplatin, and irinotecan is LINC00973. Drug resistance in GC cells is one stage where abnormal lncRNA expression has been seen. In GC, the lncRNA D63785 is overexpressed and encourages invasion, migration, and proliferation. LncD63785 is knocked down to enhance miR-422 expression, which increases doxorubicin sensitivity in GC cells. PVT1 is dysregulated and aids in the development of GC [171]. PVT1 can increase the GC cells' tolerance to 5-fluorouracil by activating BCL2. A lncRNA called MRUL is 400 kb downstream of ABCB1. MRUL is increased in multidrug-resistant GC cell lines, such as SGC7901/ADR and SGC7901/VCR. MRUL knockdown can lower ABCB1 expression levels [172]. All of these findings imply that MRUL increases the expression of ABCB1, a therapeutic target that can reverse the resistant phenotype in GC patients. Patients with GC who are cisplatin-resistant exhibit higher levels of lncRNA SNHG5 expression than do patients with GC who are cisplatin-sensitive [173]. A novel approach to treating GC that can achieve target specificity is targeted therapy based on RNA. Based on the use of small interfering RNAs and the CRISPR-associated protein 9 (CRISPR/Cas9) system in GC, lncRNAs might be made into a therapeutic target. In GC tissues and cell lines compared to other tissues, the expression of the lncRNA HOTAIR is greater. You can stop GC cells from proliferating, migrating, invading, and metastasizing by knocking down HOTAIR [174]. In GC, TINCR is overexpressed as a lncRNA. Reduction in proliferation, colony formation, and tumorigenicity can be achieved via siRNA-mediated TINCR silencing. In addition, GC tissues have higher levels of lncRNA HULC than do normal control tissues. In GC cells, HULC knockdown can increase the apoptosis that cisplatin causes. The notion that lncRNAs may develop into specific therapeutic targets for some medications in GC is supported by all of the available research [175].

According to one study, 5-FU-resistant cells, colon tumor specimens, and colon cancer cell lines, all have high levels of the lncRNA CCAT1. Colon cancer cells with high levels of CCAT1 are less sensitive to 5-FU and have lower rates of apoptosis, whereas CCAT1 knockdown has the opposite impact on colon cancer cells. These findings suggest that CCAT1 is involved in colon cancer cells' resistance to 5-FU. Vitamin D receptor (VDR) expression is decreased by lncRNA H19 via miR-675-5p, whereas H19 expression is suppressed by the VDR pathway via control of the c-Myc/ Mad-1 pathway [176]. Notably, 1,25(OH)2D3 treatment resistance is induced by H19 overexpression in colon cancer cells and mice models. In cisplatin-resistant colon cancer cells, LINC00261 is shown to be downregulated. However, LINC00261 overexpression reverses cisplatin resistance and increases the therapeutic effects of cisplatin by blocking the Wnt/ β-catenin pathway. Oxaliplatin-induced apoptosis is resistant in colon cancer cells due to CYTOR overexpression [177]. CYTOR acts as a ceRNA to control the expression of miR-193a-3p, which then upregulates the expression of the Erb-b2 receptor tyrosine kinase 4 (ERBB4). Oxaliplatin (OXA) resistance is attenuated and pAkt levels are decreased by ERBB4 inhibition. 5-FU resistance has been linked to a number of oncogenic or tumor-suppressive lncRNAs [178]. In CRC SNU-C4 and SNU-C5 cells with 5-FU resistance, one study measured the expression patterns of 90 lncRNAs using qPCR-based profiling. It found 19 differentially expressed lncRNAs and 23 lncRNAs in the 5-FU-resistant SNU-C4 and SNU-C5 cells, respectively. These lncRNAs included snaR and BACE1AS. Further evidence that snaR contributes to 5-FU resistance in colon cancer cells comes from the fact that inhibiting snaR makes cells more sensitive to the drug 5-FU [179]. ANRIL, one of onco-lncRNAs, was substantially expressed in the tissues and 5-FU-resistant GC cells BGC823/5-FU. It has also been discovered that the lncRNAs UCA1 and HULC encourage 5-FU resistance. XLOC_006753 is an upregulated lncRNA in GC patients and 5FU resistant cells SGC-7901/5-FU. It not only correlates with tumor size, metastasis, TNM stage, and a worse prognosis in GC patients, but it also contributed to 5-FU resistance by influencing cell cycle G1/S transition, apoptosis, some markers of MDR and EMT expression (180). Also, via altering the expression of the miR-23b-3p/ATG12 axis and Bcl-2, respectively, oncogenic lncRNAs MALAT1 and PVT-1 may be responsible for 5-FU resistance in GC. On the flip side, 5-FU resistance in GC can be overcome by tumor suppressor lncRNA. For instance, LncRNA LEIGC has been discovered to sensitize GC cells to 5-FU by repressing the expression of many EMT-related genes [181].

**Fig. 3:**
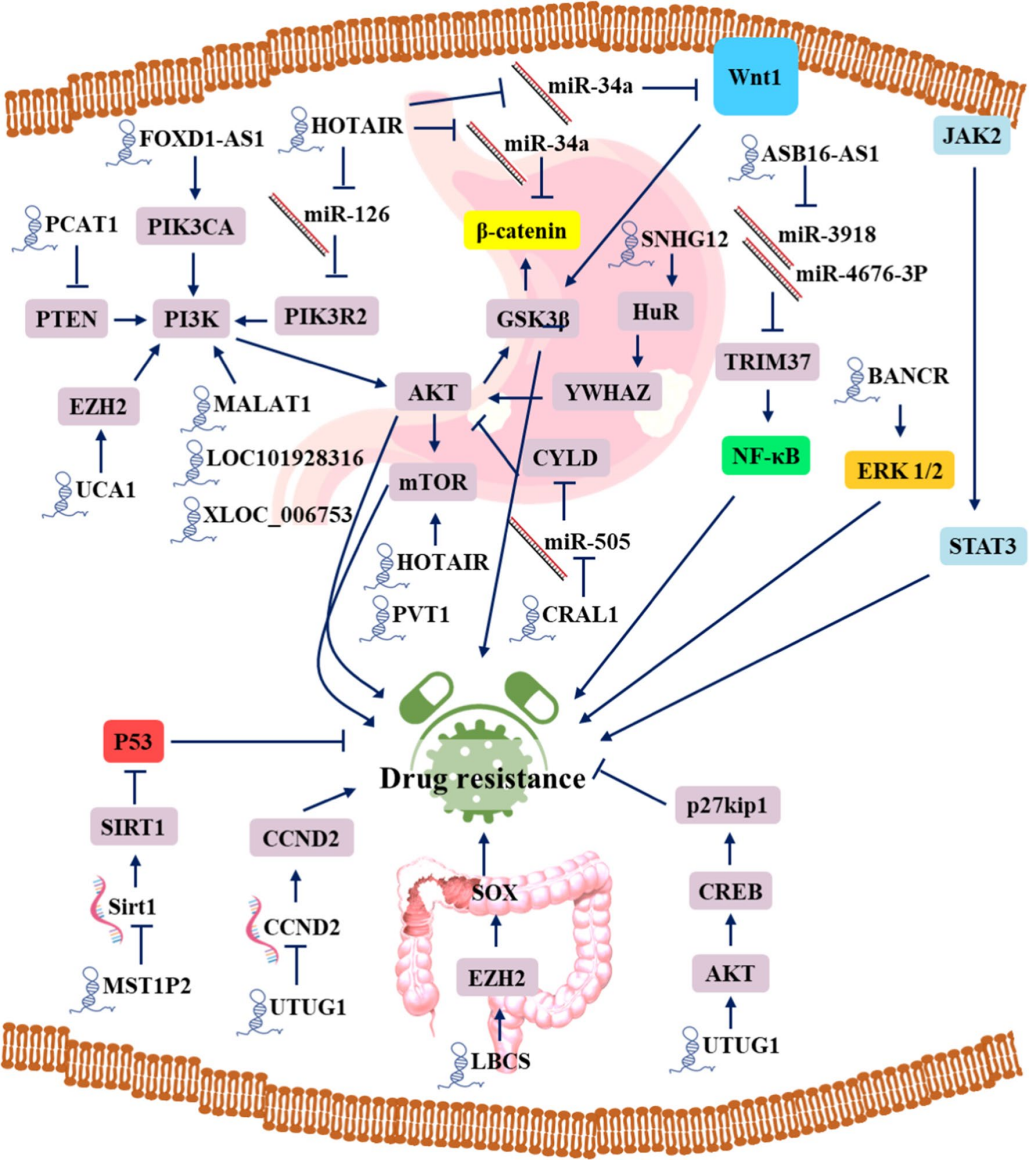
LncRNAs regulate drug resistance in gastric and colorectal cancer

Many oncogenic lncRNAs, including nuclear paraspeckle assembly transcript 1 (NEAT1), MALAT1, UCA1, and prostate cancer-associated transcript 1 (PCAT-1), as well as some tumor suppressor lncRNAs, have been shown to play a role in gastric cancer chemoresistance. Additionally, HOTAIR, CASC9, MRUL, UCA1, D63785, NEAT1, and HULC, contribute to ADR resistance [182]. For instance, the HOTAIR can increase the ADR resistance of gastric cancer cells by suppressing the expression of miR-217. It has been discovered that the lncRNA cancer susceptibility candidate 9 (CASC9), whose expression is linked to poor differentiation, invasion, and lymph node metastases in GC, increases the expression of the MDR1 protein to make gastric cancer cells resistant to ADR. It has been discovered that the lncRNA MDR-related and upregulated (MRUL), which is markedly increased in MDR SGC7901/ADR and SGC7901/VCR GC cells, promotes ABCB1 expression and, consequently, resistance to ADR and VCR [183]. It is interesting to note that lncRNA UCA1 has been demonstrated to support ADR resistance by either modulating the apoptosis-related genes PARP1 and Bcl-2 or by sponging miR-27b. The inhibition of myocyte enhancer factor-2D (MEF2D) by miR-422, which is dependent on lncRNA D63785 as ceRNA of miR-422a, has been discovered to induce ADR resistance. Additionally, HULC and NEAT1 contribute to the development of gastric cancer cells' increased ADR resistance [184]. Lately, it has been shown that ROR, which upregulates MRP1 expression in gastric cancer cells, confers resistance to ADR and VCR and is associated with poor patient outcomes. lncRNA ZFAS1 could increase the PTX resistance of SGC7901 GC cells by modifying the expression of EMT markers like E-cadherin, vimentin, and cell cycle-related proteins (cyclin B1, cyclin D1, and cyclin E) as well as the Wnt/β-catenin signaling pathway [185]. Additionally, MALAT1 targets the PTX-resistant genes miR-23b-3p and ATG12 in gastric cancer cells. Through controlling the expression of numerous genes, lncRNAs PVT1, HOTAIR, and CASC9 further increased PTX resistance in GC [180] (Fig. 3 and Table 3).

**Table 3:** lncRNAs associated with drug resistance in GC and CRC

Drug	Cancer	LncRNAS
5-FU	CRC	ANRIL, HCP5, HULC, HAND2-AS1, UCA1, XLOC_006753, ARHGAP5-AS1, MACC1-AS1, HOTTIP, PVT1, LEIGC, MALAT1, LBX2-AS1
	GC	HULC, MALAT1, HAGLR, HNF1A-AS1, ANRIL, LEIGC, UCA1, PVT-1, XLOC-006753
Cisplatin (DDP)	CRC	ANRIL, ADAMTS9-AS2, BCAR4, CRAL, ASB16-AS1, CASC2, FAM84B-AS, HOTAIR, XLOC_006753, DANCR, GHET1, HULC, ZFAS1, MALAT1, HOTTIP, PCAT-1, PVT1, SNHG5, UCA1, ROR, XIST, SNHG5,
	GC	ADAMTS9-AS2, HOTAIR, ANRIL, GHET1, AK022798, FAM84B-AS, ARHGAP5-AS1, DANCR, BCAR4, CRAL, ASB16-AS1, CAS2, PVT1, HULC, MALAT1, HOTTIP, PCAT-1, XIST, ROR, SNHG5, UCA1
OXA	CRC	GIHCG, HCP5, BLACAT1, MALAT1, DDX11-AS1, CACS15, HOTAIR, Linc00152, LUCAT1, OIP5-AS1, LncARSR, TUG1, CCAT2, MEG3, PiHL, SNHG5
	GC	MALAT1, HCP5, BLACAT1, MACCI-AS1
Platinum	GC	ASAMTS9-AS2, PCAT-1, CRAL, HOTAIR, DANCR, CASC2, SNHG5, BLACAT1, GHET1, XLOC_006753, PVT-1, AK022798, MALAT1, ANRIL, BCAR4, ZFAS1
Paclitaxel	GC	ZFAS1, CASC9, PVT-1, MALAT1, HOTAIR
Adriamycin (ADR)	GC	Xist, HOTTIP, ARHGAP5-AS1, MRUL, UCA1, HULC, CASC9, ROR, GACAT1, NEAT1

## Conclusion

In this review, we have explored the regulatory landscape of lncRNAs and their potential as diagnostic and prognostic biomarkers for GC and CRC. The role of lncRNAs in cancer development, progression, metastasis, and drug resistance has been increasingly recognized. Through complex mechanisms, lncRNAs interact with miRNAs, proteins, and other molecules to regulate gene expression at transcriptional and post-transcriptional levels.

In GC, numerous lncRNAs have been identified to play crucial roles in carcinogenesis, metastasis, prognosis, and drug resistance. Despite the progress made, several challenges hinder the clinical application of lncRNAs in GC. A comprehensive understanding of the molecular mechanisms of lncRNAs in GC is still needed. The intricate regulatory interactions between lncRNAs, other genes, and other types of ncRNAs need further exploration. Accurate measurement of lncRNAs in body fluids and tissues remains challenging due to their low abundance, ease of degradation, and instability. Furthermore, the tissue-specific expression of selected lncRNAs adds complexity to their clinical application. Similarly, in CRC, lncRNAs have emerged as predictive biomarkers for diagnosis, prognosis, and chemoresistance. Their dysregulation has been linked to epigenetic modifications, miRNA sponging, interactions with regulatory proteins, and modulation of signaling pathways. The potential of lncRNAs as therapeutic targets in CRC is promising, with the potential to increase the sensitivity of CRC cells to conventional chemotherapy drugs and targeted therapies. However, the identification and validation of specific lncRNAs that provide safe delivery systems and avoid non-specific targets remain challenging.

The knowledge gained from studying lncRNA deregulation in CRC and GC holds great potential for biomarker-oriented precision medicine approaches. It provides a foundation for developing lncRNAs as disease indices, prognostic factors, disease outcome markers, and therapeutic targets. However, further research is needed to fully understand the complex interactions between lncRNAs, ncRNAs, proteins, and genes in CRC and GC. Integrating these findings into effective therapies requires a comprehensive analysis and an integrated approach.

Moreover, the impact of lncRNAs on TME is undeniable. Dysregulation of lncRNAs can modulate the TME in CRC and contribute to tumorigenesis. While some lncRNA-miRNA-TME pathways have been elucidated. Unraveling these complexities will provide a more comprehensive understanding of the regulatory processes driving CRC and open up new therapeutic opportunities.

In this study, several lncRNAs have been identified as potential biomarkers for GC and CRC and have demonstrated significant associations with tumor differentiation, lymph node metastasis, distant metastasis, vascular invasion, overall survival, recurrence-free survival, TNM stage, and other clinical parameters. Furthermore, their dysregulation has been implicated in the modulation of critical signaling pathways involved in cell proliferation, apoptosis, EMT, and EGFR signaling.

In conclusion, lncRNAs have emerged as key players in the regulatory landscape of GC and CRC. Their roles in cancer development, progression, and drug resistance have been extensively studied. However, several challenges remain, including the need for a deeper understanding of their mechanisms, accurate measurement techniques, and tissue-specificity considerations. Despite these challenges, lncRNAs hold great promise as diagnostic and prognostic biomarkers, as well as potential therapeutic targets.

While lncRNAs have emerged as key players in the regulatory landscape of gastric and colorectal cancers, further research is warranted to unlock the full potential of lncRNAs in the management of GC and CRC patients. Investigating the specific interactions between lncRNAs, miRNAs, proteins, and other molecules will provide valuable insights into the complex regulatory networks involved in GC and CRC. Overcoming the challenges also associated with the clinical application of lncRNAs is crucial. Additional exploration of the impact of lncRNAs on the TME and finding how lncRNA dysregulation modulates the TME and contributes to tumorigenesis will provide valuable insights into the interplay between this pathway. Regardless, understanding the mechanisms through which lncRNAs modulate signaling pathways and interact with regulatory proteins will aid in the development of targeted therapies and increase the sensitivity of cancer cells to conventional chemotherapy drugs.

## Data Availability

Agree.
